# Disulfidptosis status influences prognosis and therapeutic response in clear cell renal cell carcinoma

**DOI:** 10.18632/aging.205405

**Published:** 2024-01-24

**Authors:** Weiming Deng, Zhenwei Xie, Libo Chen, Wenjin Li, Mingyong Li

**Affiliations:** 1 Department of Urology, The First Affiliated Hospital, Hengyang Medical School, University of South China, Hengyang, Hunan, China; 2 Department of Kidney Transplantation, The Third Affiliated Hospital of Sun Yat-sen University, Guangzhou, Guangdong, China; 3 Department of Endocrinology, The Affiliated Nanhua Hospital, Hengyang Medical School, University of South China, Hengyang, Hunan, China

**Keywords:** disulfidptosis, clear cell renal cell carcinoma, prognostic score, system biology, WGCNA

## Abstract

Disulfidptosis is a recently identified type of programmed cell death. It is characterized by aberrant accumulation of intracellular disulfides. The clinical implications of disulfidptosis in clear cell renal cell carcinoma (ccRCC) remain unclear. A series of bioinformatics approaches were employed to analyze ten disulfidptosis-related molecules. Firstly, the expression patterns of the disulfidptosis-related molecules were different between normal and ccRCC tissues. A comprehensive cohort of patients with ccRCC was then assembled from three public databases and subjected to cluster analysis based on disulfidptosis-related molecules. Consensus cluster analysis revealed three distinct disulfidptosis clusters. We then conducted weighted gene co-expression network analysis (WGCNA) to identify highly correlated genes. 267 hub genes were screened out through WGCNA, and three gene clusters were then determined. Finally, we identified 87 genes with prognostic value and then used them to develop a disulfidptosis scoring (DSscore) system, which was proven to independently predict survival in ccRCC. Patients in the high-DSscore group exhibited a significant survival advantage and better immunotherapeutic responses compared with those in the low-DSscore group. However, the patients in the low-DSscore group exhibited a greater degree of chemotherapeutic response. In addition, the expression of disulfidptosis-related molecules was validated by qRT-PCR, and the potential of disulfidptosis-related molecules to indicate distinct cell subtypes were validated by single-cell RNA-sequencing. In conclusion, DSscore is a promising index for predicting the prognosis and efficacy of immunotherapy in patients with ccRCC and may provide a basis for novel strategies for future studies.

## INTRODUCTION

Renal cell carcinoma (RCC) is the most common type of urinary system cancer, accounting for approximately 80% of all cases of renal malignancies [1]. In 2020, the global incidence of kidney cancer was estimated to be 431,288, with 179,368 deaths [2]. Clear cell renal cell carcinoma (ccRCC) is the most common subtype of RCC, accounting for approximately 85% of all RCC cases, and is the primary cause of mortality [3]. Advanced ccRCC is associated with a 5-year survival rate of only 12%, whereas a 5-year survival rate of up to 90% can be achieved if the disease is diagnosed at an earlier stage [4]. Unfortunately, nearly 20% of patients with ccRCC are typically not diagnosed until a late stage of disease progression [5]. Treatment for ccRCC is multimodal and includes surgery, chemotherapy, radiotherapy, targeted therapy, and immunotherapy [6]. Given the limited efficacy of radiotherapy and chemotherapy in treating ccRCC, surgery remains the primary approach; however, as many as 30% of patients experience postoperative disease recurrence within five years [7]. Hence, exploring novel prognostic models, distinguishing high-risk patients, and developing precision medicine are crucial to aiding the prognosis and treatment of ccRCC.

Disulfides represent an essential class of dynamic and redox-responsive covalent bonds and have been detected in various proteins [8]. Under oxidative stress, thiols in the cellular environment are converted to disulfides, thereby providing protection to cellular DNA against oxidative damage [9]. Dynamic thiol–disulfide homeostasis status plays critical roles in antioxidant/detoxification, apoptosis, signal transduction, and the regulation of enzymatic activity [10]. Disruption of the thiol–disulfide homeostasis status has long been considered a deleterious process in the body [11]. Recent studies involving SLC7A11^high^ cells under glucose starvation have elucidated a unique form of disulfide-related cell death distinct from apoptosis and ferroptosis, namely disulfidptosis; it is characterized by aberrant accumulation of intracellular disulfides [12]. The substantial accumulation of disulfide molecules results in anomalous disulfide bonding among actin cytoskeleton proteins, which interferes with their tissues and ultimately culminates in the collapse of the actin network and cell death [12]. Disulfide dysregulation has been implicated in the pathogenesis of various diseases, including cancer [13]. However, the relationship between disulfidptosis and the pathogenesis and clinical outcomes associated with ccRCC remains unclear. Therefore, exploring the prognostic value and molecular mechanisms of disulfidptosis is crucial for the prognosis of ccRCC and the development of therapeutic strategies against ccRCC.

In the present study, RNA sequencing and clinical information of patients with ccRCC were obtained from public databases. Subsequently, patients with ccRCC were classified into three distinct disulfidptosis clusters based on the expression patterns of 10 disulfidptosis-related molecules and similarly classified into three gene clusters based on highly correlated genes identified in weighted gene co-expression network analysis (WGCNA). A scoring system was developed to predict overall survival (OS), the response to immunotherapy, and drug sensitivity in individual patients. Most importantly, the mRNA expression of disulfidptosis-related molecules in distinct cell subtypes was validated through RNA isolation, quantitative reverse transcriptase polymerase chain reaction (qRT-PCR), and single-cell RNA sequencing (scRNA-seq) data, respectively.

## MATERIALS AND METHODS

### Data acquisition

RNA sequencing data (FPKM value) were acquired from The Cancer Genome Atlas (TCGA) and then converted into transcripts per million values. TCGA data pertaining to genome expression, variation, and correlation, were analyzed and visualized using the GSCALite website (http://bioinfo.life.hust.edu.cn/web/GSCALite/) [14]. The RNA sequencing data and clinical data of the RECA-EU (*n* = 91) and E-MTAB-1980 studies (*n* = 101) were downloaded from the International Cancer Genome Consortium (ICGC) and ArrayExpress database, respectively. Systematic batch effect correction was performed through the “ComBat” algorithm [15]. The median follow-up time was 38.75 months (range, 0–149.16 months) in this study. Additionally, the genomic and clinical data of two cohorts of patients who received immunotherapy, namely a cohort of patients with bladder cancer who received anti-programmed death ligand 1 (PD-L1) immunotherapy and a cohort of patients with metastatic melanoma who received anti-programmed death receptor-1 (PD-1) immunotherapy, were obtained from the IMvigor210 (http://research-pub.gene.com/IMvigor210CoreBiologies/) [16] and Gene Expression Omnibus (GEO, GSE78220, https://www.ncbi.nlm.nih.gov/gds/?term=GSE78220) databases, respectively [17]. Raw scRNA-seq data from seven ccRCC samples (GSE156632) sourced from the GEO database (https://www.ncbi.nlm.nih.gov/gds/?term=GSE156632) [18] was used to assess the expression levels of the disulfidptosis-related molecules in various cell subtypes. The aforementioned databases were publicly accessible and open-source. This study honors the data access policies of each database.

### Consensus clustering analysis of disulfidptosis-related molecules

We obtained and summarized data pertaining to 10 disulfidptosis-related molecules, namely GYS1, LRPPRC, NCKAP1, NDUFA11, NDUFS1, NUBPL, OXSM, RPN1, SLC3A2, and SLC7A11, from previously published literature [19, 20]. According to the expression profiles of the ten disulfidptosis-related molecules, unsupervised clustering analysis was conducted to sort patients with ccRCC into distinct disulfidptosis clusters for further analysis. The most optimal clustering number for ccRCC was then determined by evaluating the average consistency within the cluster groups. The “ConsensusClusterPlus” package in R [21] was utilized to perform every step, and this procedure criteria were selected for 50 iterations and an 80% resampling rate to ensure the robustness of the classification. Through single-sample gene set enrichment analysis (ssGSEA) [22], the enrichment score of 26 immune cells in each sample was determined and compared among the distinct clusters.

### Identification of differentially expressed genes (DEGs) and WGCNA

DEGs among the various clusters were identified using the “limma” package in R [23], with screening criteria of adjusted *P* < 0.001. The expression data of these DEGs were then employed to construct a gene co-expression network using the “weighted gene co-expression network analysis (WGCNA)” package in R [24]. Initially, both normal and ccRCC samples sourced from the TCGA database were analyzed, and the “hclust” function was used to remove outliers. Then, the optimal soft-thresholding power (β) was determined to allow scale-free topology with the “pickSoftThreshold” function. Thereafter, module eigengenes (MEs) were clustered to consolidate highly similar modules with a “mergeCutHeight” cutoff value of 0.25. To identify tumor-related modules, we calculated the correlation between the modules and clinical features (normal and tumor). The module with *P* < 0.05 was considered a gene module highly correlated with ccRCC, and the genes in the module were considered hub genes.

### Functional enrichment analysis

To determine the potential functions and enriched pathways of the hub genes in the highly correlated gene modules, Gene Ontology (GO) and Kyoto Encyclopedia of Genes and Genomes (KEGG) enrichment analyses were performed using the “Clusterprofiler” package in R [25]. *P* < 0.05 was considered to indicate statistically significant enrichment.

### Construction of the disulfidptosis scoring system

To quantify the disulfidptosis status of individual patients, we developed a scoring system, namely the DSscore system, through principal component analysis (PCA). A univariate Cox analysis of OS was performed to identify the target genes with prognostic value (*P* < 0.05). We then used the target genes with prognostic value to perform PCA, and principal components 1 and 2 were designated as characteristic scores. A DSscore formula similar to that reported in previous studies was devised: DSscore = ∑(PC1i + PC2i). On the basis of the optimal cutoff value, we classified patients into low- and high-DSscore groups for subsequent analyses. Multivariate Cox regressions were applied to detect whether the DSscore was an independent prognostic indicator. Using the “rms” package in R [26], we then developed a nomogram to quantify the 1-, 3-, and 5-year survival outcomes in ccRCC. Additionally, to evaluate the reliability of the scoring system for various subgroups stratified by clinical parameters, we performed a stratified analysis.

### Identification of immune status and tumor mutational burden (TMB) analysis

A correlation analysis was performed to illustrate the relationships between DSscores and tumor-infiltrating immune cells (TIICs). We then used the “maftools” package in R [27] to calculate TMB in ccRCC samples sourced from TCGA. The variance and correlation of TMB across the different DSscore groups were investigated through the Wilcoxon test and Spearman correlation analysis.

### Immunotherapeutic efficacy and drug susceptibility analysis

The data of IMvigor210 and GSE78220 cohorts were used to evaluate the ability of DSscores to predict the response to immune checkpoint blockade treatment. Survival analyses were conducted using Kaplan-Meier survival curves and the log-rank test (*P* < 0.05). To determine the responses to chemotherapeutics, we determined the semi-inhibitory concentration (IC50) values of various drugs in the low- and high-DSscore groups using the “oncoPredict” package in R [28]. The differences in IC50 values between DSscore groups were examined with Wilcoxon signed-rank tests (*P* < 0.01).

### Analysis of mRNA expression levels of disulfidptosis-related molecules using qRT-PCR

A total of 10 paired samples of ccRCC and adjacent non-tumorous tissues were obtained from the First Affiliated Hospital of the University of South China. Total RNA was extracted from the tissues using TRIzol (Invitrogen, Carlsbad, CA, USA). Reverse transcription was conducted using the HiScript RT Super Mix (Vazyme Biotech Co., Ltd., China). qRT-PCR was performed using the CFX96 Real-Time System (Bio-Rad, Hercules, CA, USA). The PrimeScript RT Reagent Kit (TaKaRa, Tokyo, Japan) was used to synthesize complementary DNA. qPCR was performed using the SYBR Green Assay Kits (TaKaRa) on a CFX96 Real-Time PCR Detection System (Bio-Rad, Hercules, CA, USA). The PCR program was as follows: an initial melting step at 95°C for 30 s followed by 40 cycles of denaturation at 95 °C for 5 s, annealing at 60°C for 30 s, and a final extension at 95°C for 10 s. Relative gene expression levels were determined using the 2^−ΔΔCt^ method and normalized to that of GAPDH. The sequences of primers used for qRT-PCR are listed in Supplementary Table 1. Written informed consent was obtained from all patients. This study was conducted according to the guidelines of the Declaration of Helsinki and was approved by the Ethical Review Committee of the First Affiliated Hospital of the University of South China.

### Validation of the disulfidptosis-related molecules by scRNA-seq analysis

Furthermore, the scRNA-seq count matrix of the GSE156632 dataset was obtained to illustrate the expression of disulfidptosis-related molecules in various cell subtypes [18]. The matrix was converted into Seurat object using the “Seurat” package in R [29], and quality control was conducted. Briefly, cells with “nFeature” less than 200 and “percent.mt” less than 20% were filtered out. The data were normalized, and PCA was performed for the top 1,500 genes that exhibited highly variable characteristics using the “NormalizeData” function in Seurat. The “FindNeighbors” and “FindClusters” functions in the “seurat” package were used to execute unsupervised cluster analysis. The “FindAllMarkers” function was used to compare the differences in gene expression between a cluster and all other clusters. The “SingleR” package in R [30] was used to annotate the cell subtypes of the obtained cell clusters with the reference dataset “HumanPrimaryCellAtlasData” to elucidate the expression levels and the correlations among the disulfidptosis-related molecules.

### Statistical analysis

All statistical analyses were performed using R, and *P* < 0.05 (two-tailed) was considered to indicate statistical significance in the absence of special annotation.

### Data availability statement

The datasets analyzed in this study can be found in TCGA (http://www.cancer.gov/tcga), ICGC (https://dcc.icgc.org/projects/RECA-EU), ArrayExpress (https://www.ebi.ac.uk/biostudies/arrayexpress/), IMvigor210 (http://research-pub.gene.com/IMvigor210CoreBiologies/), and GEO (https://www.ncbi.nlm.nih.gov/gds/, GSE78220, and GSE156632) databases.

## RESULTS

### Genetic characteristics and transcriptional variations of 10 disulfidptosis-related molecules

**Figure 1:**
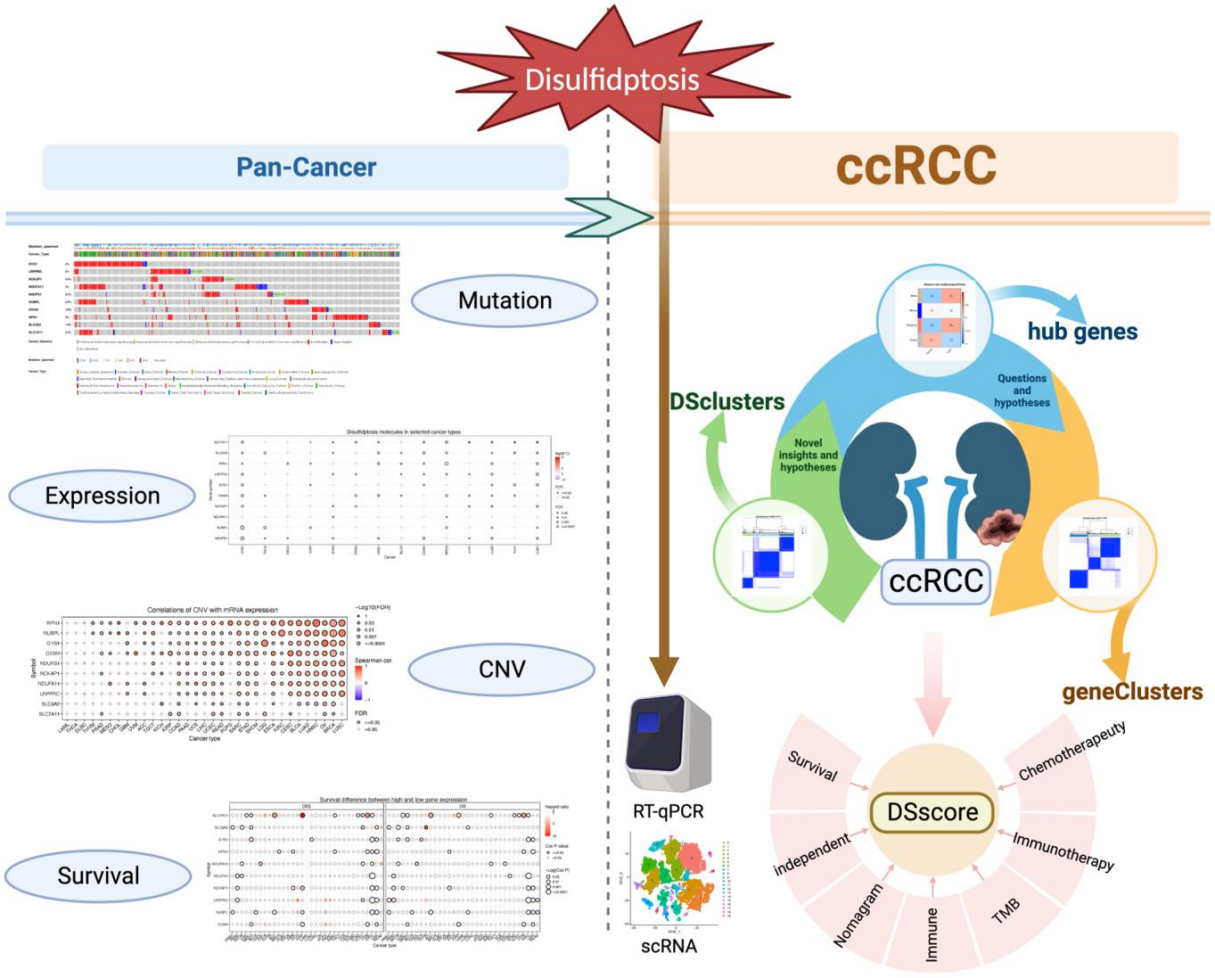
The flow of this study.

**Figure 2:**
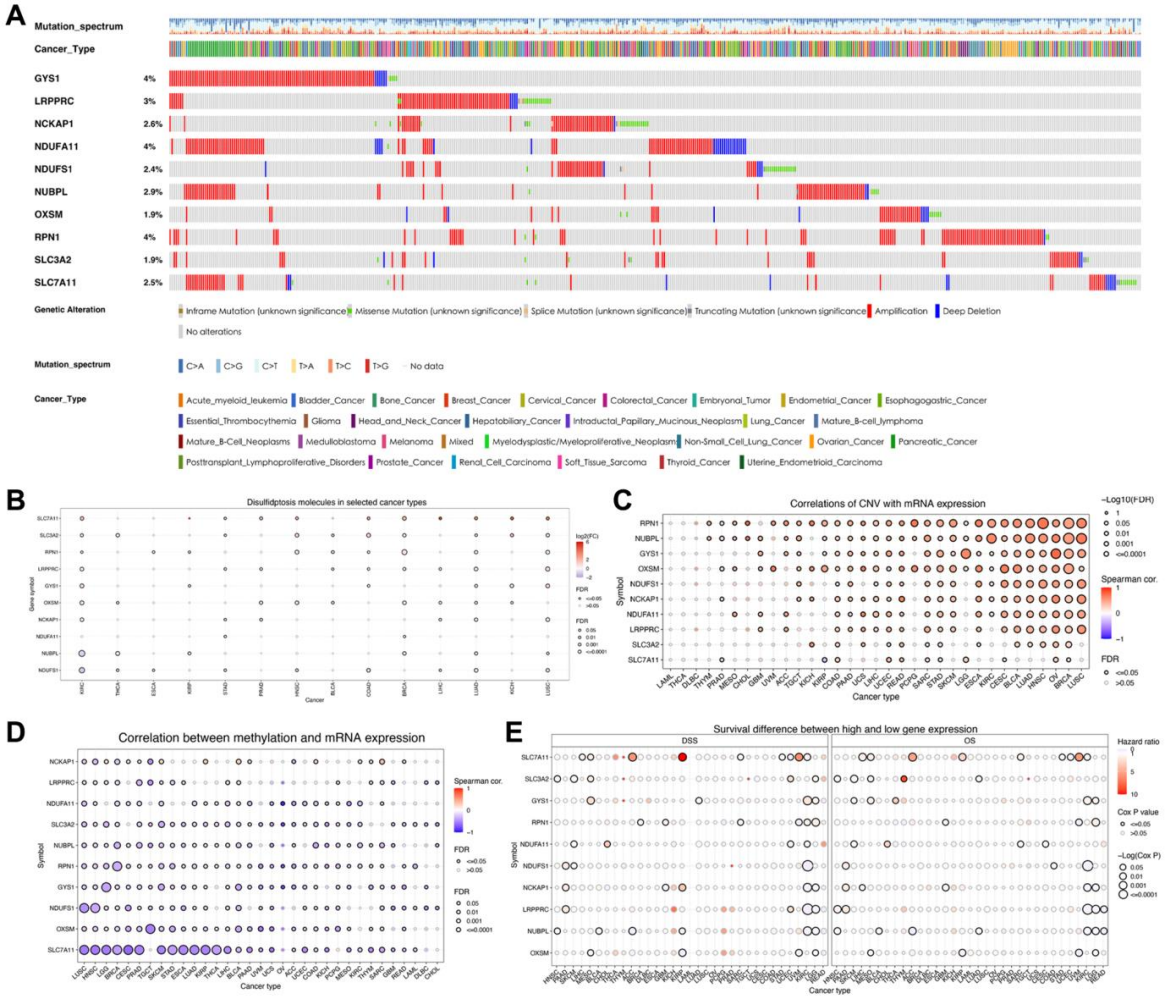
**Variations in the expression of disulfidptosis-related molecules as revealed by the pan-cancer analysis.** (**A**) A waterfall diagram illustrates the somatic mutations pertaining to the ten disulfidptosis-related molecules in pan-cancer using the cBioPortal database. (**B**) The expression levels of disulfidptosis-related molecules in 14 cancer types. (**C**) A bubble chart depicting the correlations between CNV and mRNA expression levels. (**D**) A bubble chart depicting the correlations between methylation and mRNA expression levels. (**E**) Differences in survival between patients exhibiting low and high expression of genes.

The flow of this study is shown in Figure 1. To investigate the mutations in disulfidptosis-related molecules in ccRCC, we obtained data pertaining to 2,683 samples from TCGA and ICGC pan-cancer databases through the cBioPortal server. Then, a waterfall chart was generated to illustrate the frequency of somatic mutations in the ten disulfidptosis-related molecules across pan-cancer tissues (Figure 2A). We observed that the mutations in GYS1, NDUFA11, and RPN1 exhibited the highest frequency (4%), with the predominant mutation type being amplification mutation. Subsequently, we compared the mRNA expression levels of the ten disulfidptosis-related molecules across 14 types of cancer (more than ten paired normal and tumor samples). The results revealed that SLC7A11, SLC3A2, RPN1, LRPPRC, and GYS1 were highly expressed in several cancer tissues (Figure 2B). Notably, with the exception of NDUFA11, all disulfidptosis-related molecules showed differential expression in both ccRCC and lung adenocarcinoma. In addition, we observed that genetic variation plays a critical role in the expression of disulfidptosis-related molecules. A bubble plot showed that the levels of copy number variations (CNV) were positively associated with mRNA expression in most types of cancer (Figure 2C). Analysis of the frequency of CNV changes revealed that CNV changes were common in all ten disulfidptosis-related molecules. OXSM, NDUFA11, and SLC7A11 showed the highest CNV frequency, primarily heterozygous CNV amplification and deletions (Supplementary Figure 1A). However, homozygous CNV was less prevalent (Supplementary Figure 1B, 1C). Compared to those in the normal samples, the changes in gene methylation in most cancer specimens were significantly downregulated (Supplementary Figure 1D). Moreover, a significantly negative correlation was observed between mRNA expression and gene methylation in multiple tumors (Figure 2D). These results provide comprehensive evidence that gene expression profiles of disulfidptosis-related molecules in individual tumors are highly heterogeneous, indicating a strong association between aberrant expression and genomic variance.

**Figure 3:**
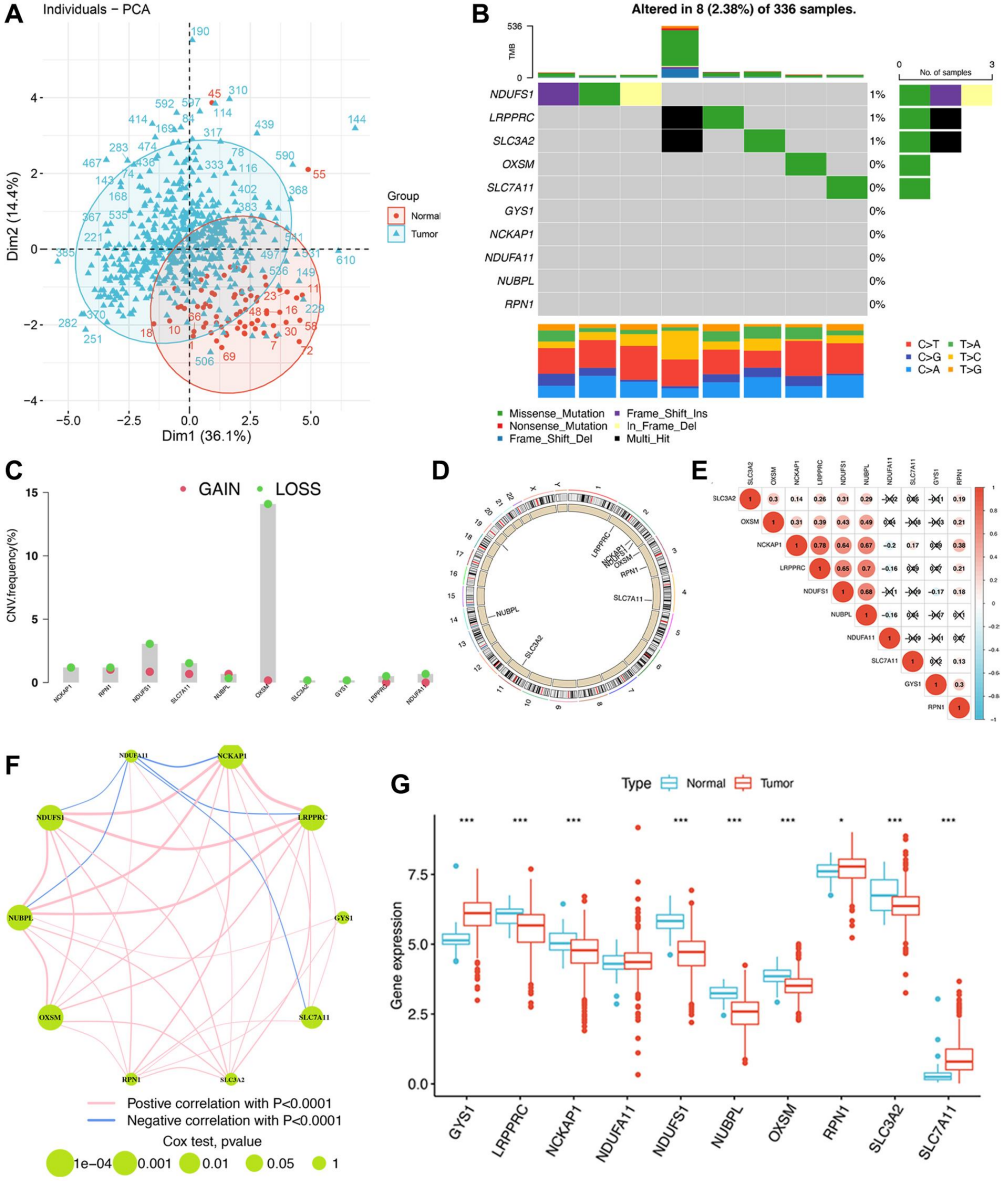
**The genomic and transcriptomic landscape of disulfidptosis-related molecules in ccRCC.** (**A**) Principal component analysis shows a marked difference in the transcriptome between normal and ccRCC samples. (**B**) The mutation landscape of disulfidptosis-related molecules. (**C**) The CNV frequency of disulfidptosis molecules. (**D**) The location of disulfidptosis-related molecules on the chromosome. (**E**) Pearson correlation analysis of disulfidptosis-related molecules. (**F**) The expression of and the interactions between disulfidptosis-related molecules. (**G**) The differences in mRNA expression levels of disulfidptosis-related molecules between normal and ccRCC samples. ^*^P < 0.05, ^***^P < 0.001.

We then analyzed the correlation between the expression of disulfidptosis-related molecules and both clinical outcomes and the pathologic stage of pan-cancer tissues. Analyses of disease-specific survival and OS revealed that the prognosis of various patients with cancer, particularly those with ccRCC, is influenced by the expression levels of disulfidptosis-related molecules (Figure 2E). In addition, several disulfidptosis-related molecules showed a notable correlation with the stage of ccRCC while exhibiting a weak correlation with that of other tumors (Supplementary Figure 1E). Therefore, we further investigated disulfidptosis in ccRCC in this study. PCA revealed that the gene expression profiles of disulfidptosis-related molecules were different between the control and ccRCC samples obtained from the TCGA database (Figure 3A). Among the 336 ccRCC samples, eight samples exhibited mutations (with a frequency of 2.38%) in three disulfidptosis-related molecules, namely NDUFS1, LRPPRC, and SLC3A2 (Figure 3B). Further analysis of CNV mutations revealed that most of the disulfidptosis-related molecules exhibited a decrease in CNV frequency (Figure 3C). Figure 3D depicts the chromosomal locations of the disulfidptosis-related molecules. Furthermore, we analyzed the interactions among the ten disulfidptosis-related molecules by Spearman’s correlation analysis (Figure 3E). NCKAP1 exhibited the strongest positive correlation with LRPPRC. WGCNA results revealed the close relationships among the ten disulfidptosis-related molecules in patients with ccRCC (Figure 3F). We also found that most of the disulfidptosis-related molecules (9/10, 90%) were differentially expressed between normal and tumor tissues (Figure 3G). Among them, GYS1 and SLC7A11 were highly upregulated in ccRCC tissues, whereas LRPPRC, NCKAP1, NDUFA11, NDUFS1, NUBPL, OXSM, RPN1, and SLC3A2 were downregulated in ccRCC tissues. Taken together, these results showed that the crosstalk among the disulfidptosis-related molecules might play indispensable roles in the progression of ccRCC.

### Consensus clustering analysis of disulfidptosis-related molecules

**Figure 4:**
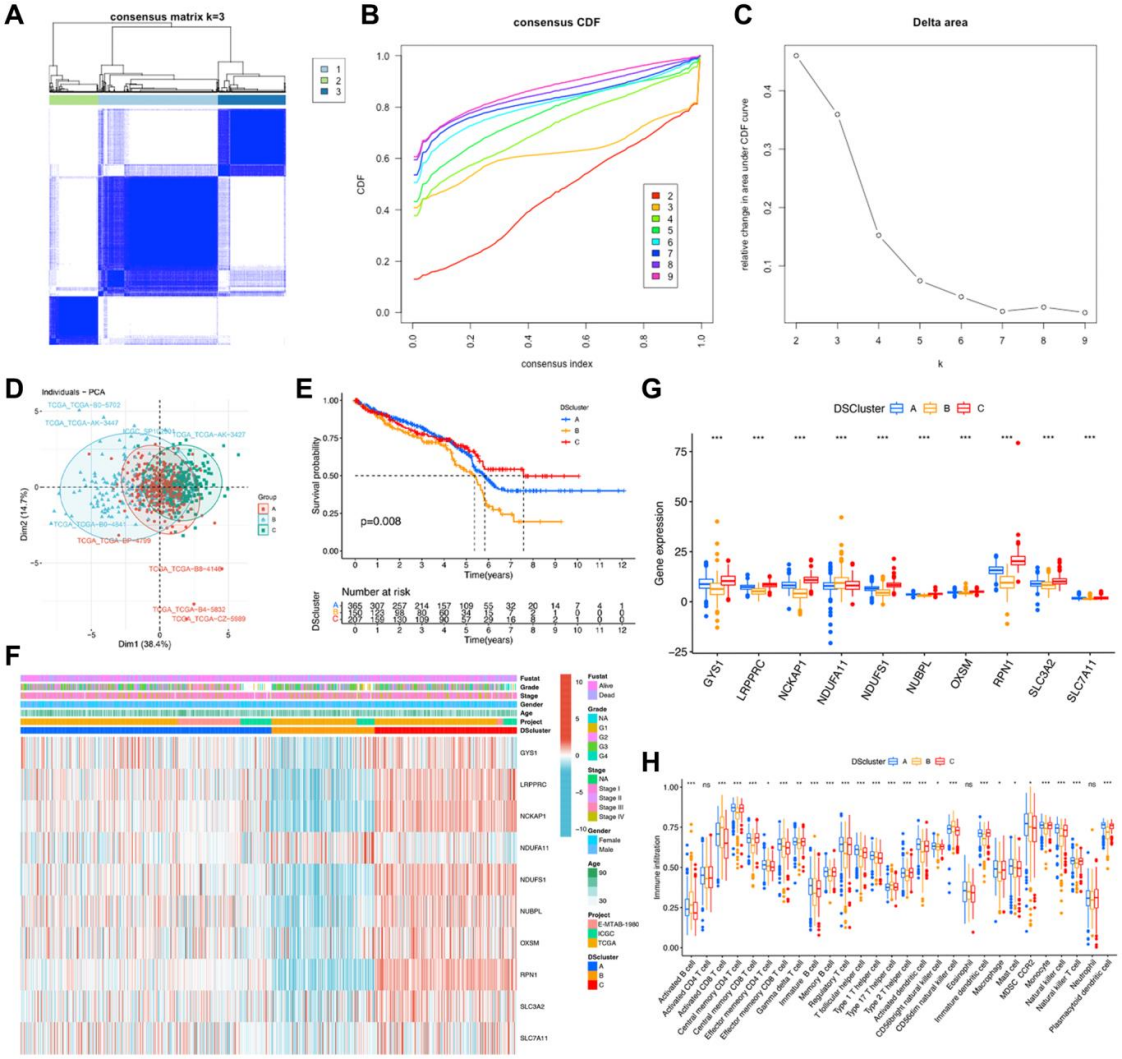
**Identification of disulfidptosis-related clusters in ccRCC.** (**A**) A color-coded heat map of the consensus matrix for k = 3. (**B**) Cumulative distribution function (CDF) curve of unsupervised clustering when k = 2–9. (**C**) Relative change in area under the CDF curve when k = 2–9. (**D**) Principal component analysis shows notable differences in the transcriptome among the three disulfidptosis clusters. (**E**) Kaplan–Meier survival curves corresponding to the various disulfidptosis clusters. (**F**) A heat map of disulfidptosis-related molecules and clinicopathological features of patients in the three disulfidptosis clusters. (**G**) The expression of the ten disulfidptosis-related molecules in the three disulfidptosis clusters. (**H**) The abundance of 28 types of infiltrating immune cells in the three disulfidptosis clusters. ^*^P < 0.05, ^**^P < 0.01, ^***^P < 0.001. Abbreviation: ns: no significant.

To comprehensively investigate the potential mechanisms of disulfidptosis-related molecules in ccRCC, we obtained 722 ccRCC samples from three datasets. Consensus clustering of patients with ccRCC was conducted to determine the different patterns of the expression of 10 disulfidptosis-related molecules. The k-median clustering algorithm revealed three disulfidptosis clusters (Figure 4A–4C, Supplementary Table 2). Accordingly, the patients with ccRCC were classified into disulfidptosis cluster A (DScluster A, *n* = 365), disulfidptosis cluster B (DScluster B, *n* = 150), and disulfidptosis cluster C (DScluster C, *n* = 207). The accuracy of this classification method was validated by PCA (Figure 4D). Survival analysis of the three disulfidptosis clusters revealed that DScluster B exhibited the worst prognosis (*P* = 0.008) (Figure 4E). Notably, we found a marked crossover in the survival curves of DSclusters A and C. We also compared the clinicopathological features and the expression levels of disulfidptosis-related molecules among the three clusters by constructing a heat map (Figure 4F). The majority of the disulfidptosis-related molecules were significantly highly upregulated in DScluster C, whereas they were the most downregulated in DScluster B, and these differences were statistically significant (Figure 4G). To determine the impact of the disulfidptosis-related molecules on the tumor microenvironment (TME) of ccRCC, we then performed the ssGSEA to assess the relative degree of infiltration of 28 different immune cell types. The results showed remarkable differences in the abundances of most immunocytes among the three DSclusters of ccRCC (Figure 4H).

### Identification of highly correlated gene modules in TCGA–ccRCC

**Figure 5:**
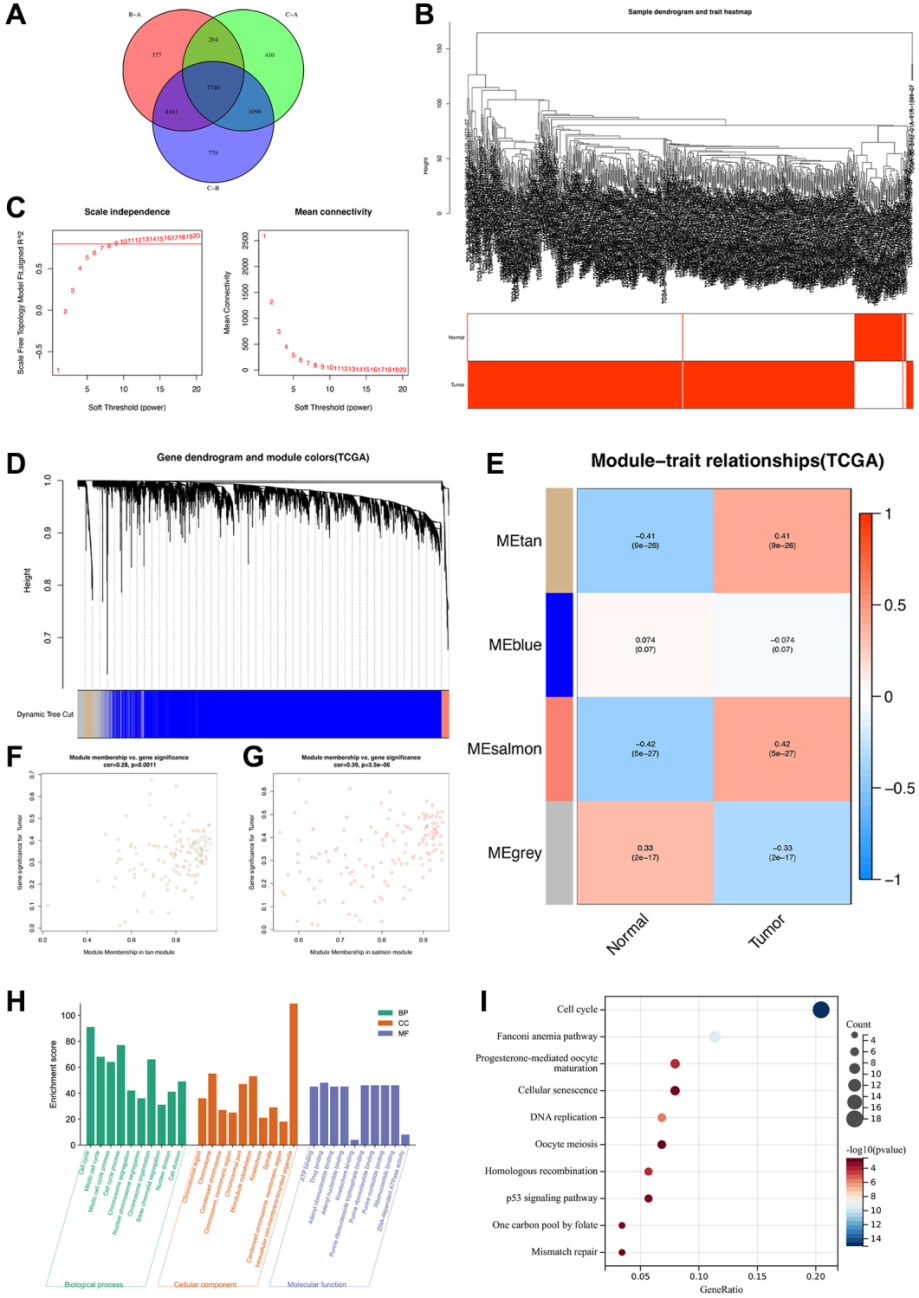
**Identification of highly correlated gene modules in TCGA-ccRCC.** (**A**) A Venn diagram of 7740 disulfidptosis cluster-related genes. (**B**) Clustering dendrogram of samples corresponding to clinical traits. (**C**) Determination of the scale-free fit index for various soft-thresholding powers (β). (**D**) A dendrogram of genes was clustered based on a dissimilarity measure (1-TOM). (**E**) A heat map illustrating the correlations among the module eigengenes and clinical traits (normal and tumor). (**F**) Gene correlation scatter plot of the tan module. (**G**) Gene correlation scatter plot of the salmon module. (**H**) GO enrichment analysis and (**I**) KEGG pathway enrichment analysis of genes in tan and salmon modules.

To determine the heterogeneity in different disulfidptosis clusters, we identified DEGs among the clusters using the “limma” package in R. A total of 7,740 disulfidptosis cluster-related genes were identified (Figure 5A). Subsequently, to identify the tumor-related genes, we performed WGCNA of the 7,740 genes and identified the highly correlated gene modules of hub genes. Initially, 72 normal and 539 ccRCC tissues from the TCGA database were clustered, and anomalous samples were eliminated by establishing a threshold (Figure 5B). The soft threshold power was then determined to be 8 for satisfying a scale-free network distribution (scale-free R^2^ = 0.9) (Figure 5C). Similar gene modules were then identified and merged, and eventually, four co-expression modules (tan, blue, salmon, and gray) were obtained (Figure 5D). Subsequently, to identify the modules that exhibited the strongest association with ccRCC, we examined the correlations between ME values and clinical features (normal vs. tumor). The results showed that the salmon (R = 0.42, *P* = 5e-27) and tan (R = 0.41, *P* = 9e-26) modules exhibited statistically significant positive correlations with ccRCC (Figure 5E). In addition, Pearson’s correlation analysis of the genes in the tan (Figure 5F) and salmon (Figure 5G) modules revealed that they were highly correlated. Thus, a total of 267 genes from the aforementioned modules were designated as hub genes and subsequently analyzed.

To determine the biological functions and pathways associated with the 267 hub genes, we performed functional enrichment analyses. GO analysis revealed that these genes were significantly enriched in biological process terms associated with cell cycle, chromosome organization, and mitotic cell cycle process (Figure 5H). Moreover, KEGG analysis revealed that these genes were notably enriched in several pathways, such as cell cycle, cellular senescence, and the p53 signaling pathway (Figure 5I), which are associated with the development of ccRCC. Overall, these 267 genes were determined to be highly correlated with the development and progression of ccRCC.

### Identification of gene clusters based on the hub genes

**Figure 6:**
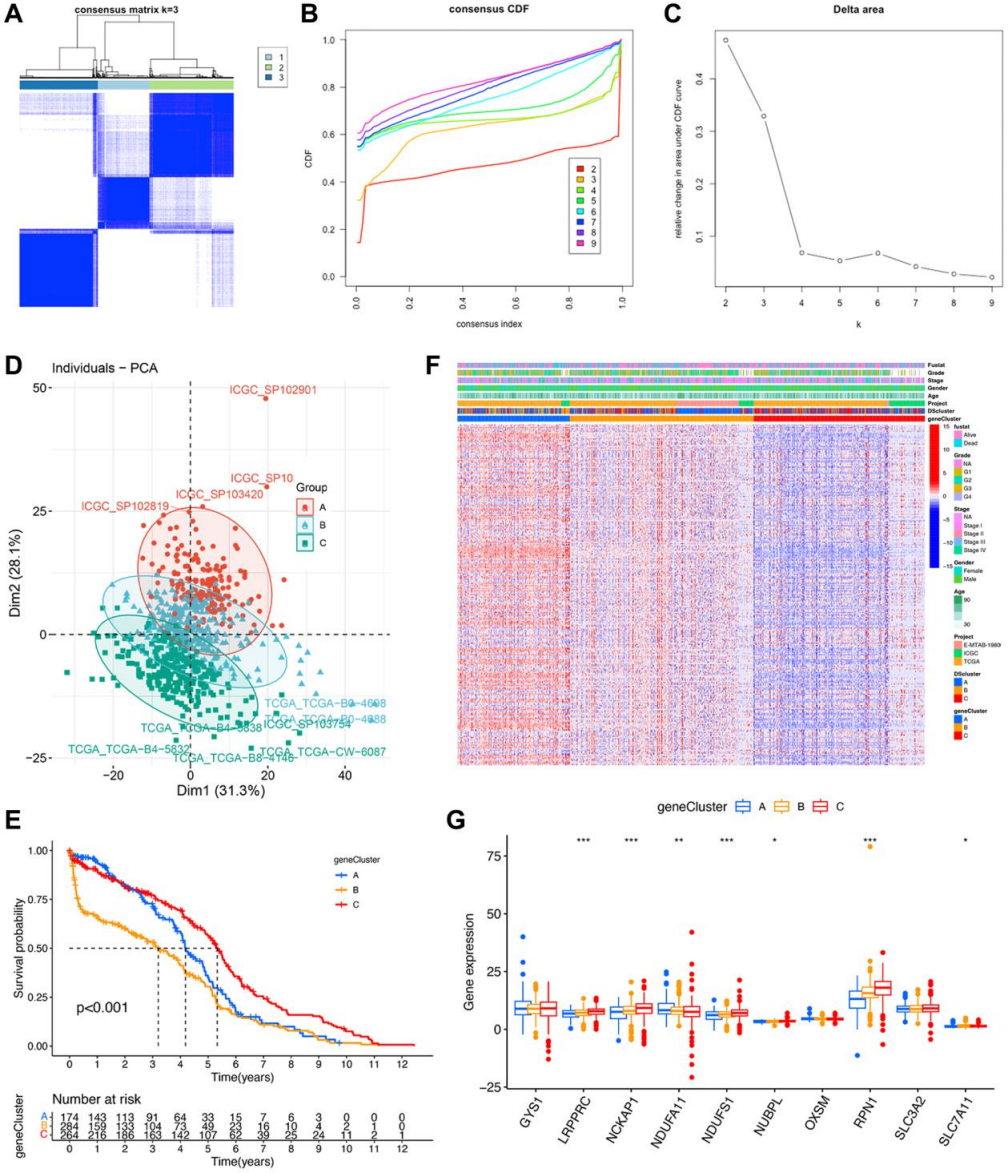
**Identification of disulfidptosis gene clusters in ccRCC.** (**A**) A color-coded heat map of the consensus matrix for k = 3. (**B**) Cumulative distribution function (CDF) curve of unsupervised clustering when k = 2–9. (**C**) Relative changes in area under the CDF curve when k = 2–9. (**D**) Principal component analysis showed a marked difference in the transcriptome between the three disulfidptosis gene clusters. (**E**) Kaplan–Meier survival curves of different disulfidptosis gene clusters. (**F**) Heat map of hub genes and clinicopathological features of patients in the three disulfidptosis gene clusters. (**G**) Expression of 10 disulfidptosis molecules in the three disulfidptosis gene clusters. ^*^P < 0.05, ^**^P < 0.01, ^***^P < 0.001.

To further validate the regulation mechanism, we then identified different disulfidptosis-related gene clusters based on the expression of these 267 genes by WGCNA. The unsupervised clustering algorithm also classified patients with ccRCC into three distinct disulfidptosis-related gene clusters, namely geneCluster A, B, and C, consistent with the clustering based on disulfidptosis-related molecules (Figure 6A–6C) (Supplementary Table 2). This classification method was also validated by PCA (Figure 6D). Moreover, geneCluster C exhibited a particularly significant survival advantage, whereas geneCluster B exhibited the poorest prognosis (Figure 6E). A heat map revealed that most of the genes exhibited a gradual decrease in expression from geneCluster A to geneCluster C (Figure 6F). Seven disulfidptosis-related molecules were also significantly differentially expressed among the three geneClusters (Figure 6G), consistent with the results pertaining to the DSclusters.

### Construction of a disulfidptosis scoring system

**Figure 7:**
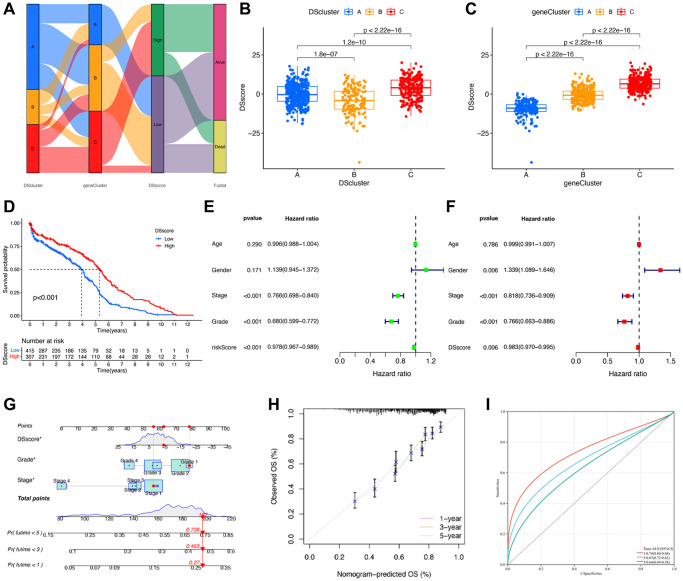
**Construction of disulfidptosis score system.** (**A**) Sankey plot of DScluster, geneCluster, DSscore, and survival status. (**B**) The differences in the DSscore among the three DSclusters. (**C**) The differences in the DSscore among the three disulfidptosis geneClusters. (**D**) Kaplan–Meier survival curves of low- and high-DSscore groups. (**E**) Univariate and (**F**) Multivariate Cox regression analyses of the DSscore and clinical features to determine prognostic value. (**G**) A comprehensive nomogram for predicting the survival probability of patients with ccRCC. (**H**) The calibration curve of the nomogram. (**I**) ROC curve of the nomogram. ^*^P < 0.05.

A total of 87 genes were identified as the potential prognostic genes in the tan and salmon modules in univariate Cox regression analysis. To further depict an individual’s disulfidptosis status, we established a risk score system, namely the DSscore system, via PCA of the 87 disulfidptosis cluster-related genes (Supplementary Table 2). As shown in the alluvial diagram that depicts the changes in the attributes of DScluster, geneCluster, DSscore, and survival status (Figure 7A), the DSscores were significantly different among the three different DSclusters. In detail, DScluster C exhibited the highest DSscore, followed by DSclusters A and B, in that order (Figure 7B). Significant differences in DSscores were also observed among the geneClusters (Figure 7C). The patients were then divided into two (low-DSscore and high-DSscore groups) according to a cutoff DSscore. The low-DSscore group exhibited considerably lower survival rates compared with the high-DSscore group (Figure 7D). Univariate and Multivariate analysis confirmed that stage, grade, and DSscore were an independent prognostic factor of OS in ccRCC (Figure 7E, 7F). A nomogram that included three independent clinical factors were then successfully established as a clinically applicable quantitative method for predicting individual OS (Figure 7G). The calibration curve confirmed the nomogram had optimal goodness of fit between the predicted and actual OS probabilities (Figure 7H). The ROC curve showed that the nomogram had a better discrimination ability (Figure 7I). Moreover, stratification analyses were performed to validate whether the DSscore retained its predictive ability in distinct subgroups. The results showed that compared with those with low DSscores, patients with high DSscores exhibited longer OS in all the subgroups, including age (age ≤60 and age >60), sex (female and male), stage (stage I and II and stage III and IV), and grade (grades 1–2 and grades 3–4) (Supplementary Figure 2). Collectively, these results indicated the feasibility of utilizing the DSscore-based classification method for predicting the OS of patients with ccRCC.

### DSscore is a predictor of the response to immunotherapy

**Figure 8:**
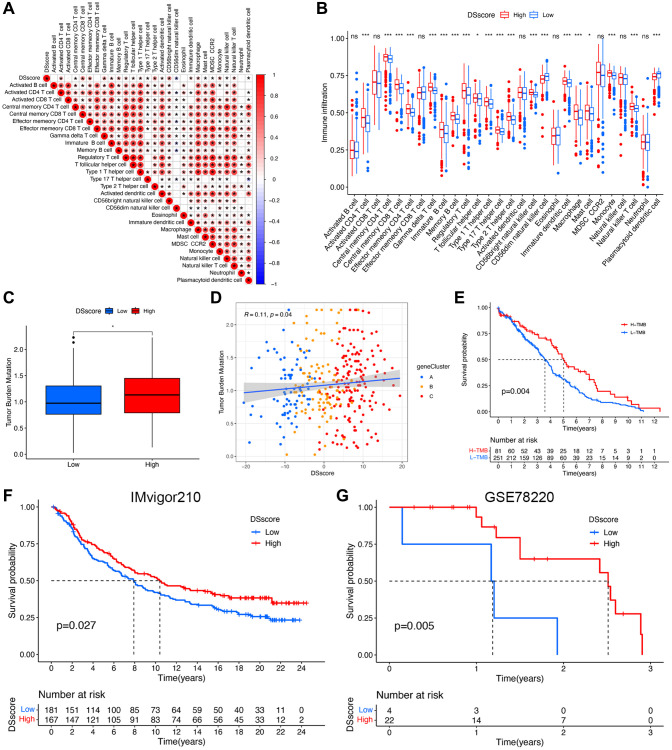
**DSscore predicts the response to immunotherapy.** (**A**) The correlation between DSscore and immune cell infiltration. (**B**) The differences in immune cell infiltration between the two DSscore groups. (**C**) The comparison of TMB between the two DSscore groups. (**D**) Correlation analysis of DSscore and TMB value. (**E**) Kaplan–Meier survival curves of the low- and high-TMB groups. (**F**) Kaplan–Meier survival curves of the low- and high-DSscore groups (IMvigor210 cohort). (**G**) Kaplan–Meier survival curves of low- and high-DSscore groups (GSE78220 cohort). ^*^P < 0.05, ^***^P < 0.001. Abbreviation: ns: no significant.

We evaluated the correlation between DSscore and various immune cells and found a positive correlation between the DSscore and 21 of 28 immune-related cell infiltrates (Figure 8A), indicating a positive regulation of the immune reaction in patients with high DSscores. This finding was further emphasized by the difference in the immune cell profiles between the two DSscore groups (Figure 8B). Statically significant differences in the levels of TMB were observed between the two DSscore groups, and a positive correlation was observed between DSscores and TMB values. TMB analysis revealed that the TMB values were higher in the high-DSscore groups than those in the low-DSscore groups (Figure 8C). Further analyses revealed that TMB values were positively associated with DSscore ratings (Figure 8D). Survival analysis revealed that patients in the high-TMB group exhibited longer OS than that exhibited by patients in the low-TMB group (Figure 8E), suggesting that the prognosis of patients with ccRCC was influenced by TMB. Given that the inhibition of immunologic checkpoints has emerged as an unprecedented anticancer treatment, we then evaluated the prognostic value of DSscore in two cohorts who received immune checkpoint therapy (IMvigor210 cohort, anti-PD-L1 therapy; GSE78220 cohort; anti-PD-1 therapy). The patients in the IMvigor210 cohort and GSE78220 cohort were categorized into low- and high-DSscore groups. Patients in the high-DSscore group exhibited significantly longer OS compared with those in the low-DSscore group in both the IMvigor210 (*P* = 0.027, Figure 8F) and GSE78220 cohorts (*P* = 0.005, Figure 8G), suggesting the patients in the high-DSscore group exhibited superior immunotherapeutic repones. This finding was consistent with the finding that the high-TMB group was more likely to benefit from immunotherapy.

### DSscore is a predictor of drug sensitivity

To further examine the clinical utility of DSscore for precise ccRCC treatment, we evaluated the therapeutic efficacy of several commonly used chemotherapeutics in various DSscore groups. The results of analyses of IC50 values showed that patients with low DSscores were more responsive to MIRA-1, navitoclax, linsitinib, and PRIMA-1MET, whereas they were less sensitive to KU-55933 (Supplementary Figure 3).

### Validation of the disulfidptosis-related molecules using RT-qPCR and scRNA-seq analysis

On the basis of the DSscore, we validated that all ten disulfidptosis-related molecules were closely linked with the OS of patients with ccRCC using survival curves (Supplementary Figure 4). Of the disulfidptosis-related molecules, GYS1 and SLC7A11 showed elevated expression and were associated with poor prognosis, whereas the remaining eight genes correlated with favorable prognosis. The expression levels of these molecules were quantified in human ccRCC tissues and pair-matched adjacent normal tissues (10 pairs) by RT-qPCR (Supplementary Figure 5). We found that the expression of GYS1 and SLC7A11 were upregulated, while those of NCKAP1, NDUFS1, NUBPL, OXSM, RPN1, and SLC3A2 were downregulated in ccRCC tissues compared with those in the para-carcinoma tissues. The expression levels of LRPPRC and NDUFA11 were decreased in ccRCC tissues; however, the differences were not statistically significant, possibly because of the small sample sizes.

**Figure 9:**
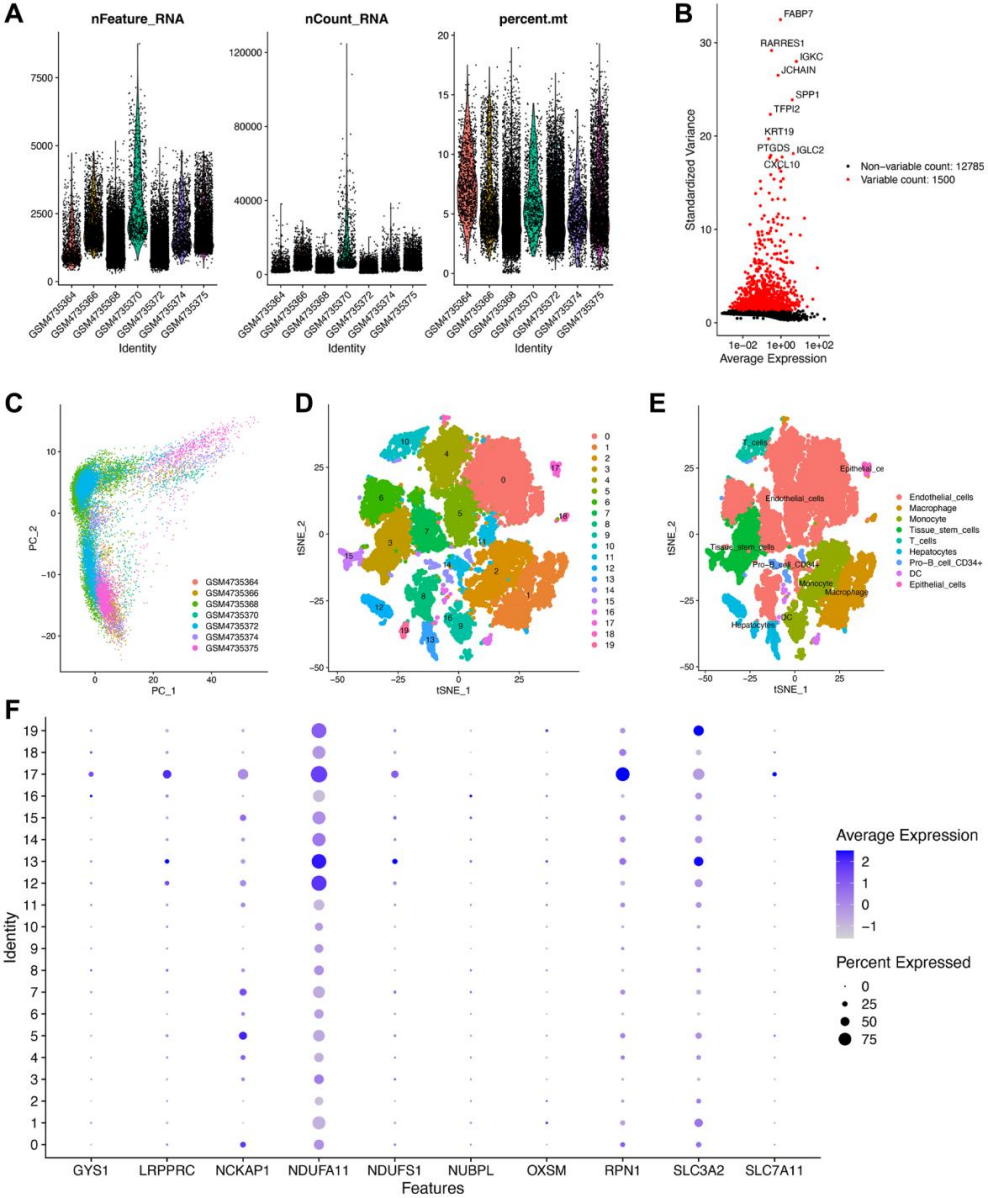
**Validation of the expression of the disulfidptosis-related molecules using scRNA-seq data.** (**A**) Following quality control and normalization, (**B**) detection of highly variable genes across the cells using a volcano plot, the top 10 genes are marked. (**C**) PCA based on scRNA-seq data from seven ccRCC samples. (**D**) tSNE algorithm classified cells into 20 clusters. (**D**) The cell types of the various clusters. (**E**) In total, 20 cell clusters were annotated into nine cell subtypes using the tSNE algorithm. (**F**) The expression level of the disulfidptosis-related molecules in each cell subtype.

Finally, we analyzed scRNA-seq data from the GSE156632 cohort to determine whether the disulfidptosis-related molecules could be used to discriminate between different cell subtypes. Following quality control and normalization, a total of 21,671 cells from seven ccRCC samples were subsequently analyzed (Figure 9A). Additionally, among the 12,785 genes across the cells, 1,500 genes that exhibited high variability were depicted in a volcano plot (Figure 9B). The distribution of ccRCC cells among different samples was elucidated by PCA (Figure 9C). ccRCC cells were then successfully clustered into 20 clusters using the tSNE algorithm based on nonlinear dimension reduction (Figure 9D). Expression analysis revealed 2,474 marker genes for 20 cell clusters, and the top 10 genes of each cluster were visualized in a heat map (Supplementary Figure 6). Subsequently, these 20 cell clusters were categorized into nine cell subtypes. In detail, clusters 0, 4–8, and 11 were endothelial cells; clusters 1 and 18 were macrophages; clusters 2 and 9 were monocytes; clusters 3 and 15 were tissue stem cells; clusters 12, 13, and 19 were fibroblast; cluster 10 was T cells; cluster 14 was CD34+ pro-B cells; cluster 16 was dendritic cells; cluster 17 was epithelial cells (Figure 9E). In epithelial cells, GYS1, LRPPRC, NCKAP1, RPN1, and SLC7A11 were highly expressed (Figure 9F). In fibroblast, NDUFA11, NDUFS1, and SLC3A2 were highly expressed. However, NUBPL and OXSM did not exhibit discernable expression patterns. These results validated the potential of disulfidptosis-related molecules in distinguishing between cell subtypes.

## DISCUSSION

ccRCC is considered the most devastating tumor of the urinary system owing to its high incidence of recurrence and metastasis, as well as its insensitivity to chemoradiotherapy [31]. Previous studies have primarily focused on a single experimental study for ccRCC. However, few studies have reported satisfactory results. The extraction and identification of genetic data have garnered increasing attention in light of advancements in high-throughput sequencing and bioinformatics analysis. Programmed cell death (PCD) has been reported to play an essential role in ccRCC progression [32]. Many recent studies have developed prognostic signatures associated with PCD, such as cuproptosis [33], ferroptosis [34], and pyroptosis [35], for clinical guidance in ccRCC. Nevertheless, to date, the functions or signatures and the regulatory mechanisms of disulfidptosis in ccRCC remain unclear.

Disulfidptosis, a novel form of cell death reported in 2023 by Liu et al., has attracted the attention of researchers worldwide [12, 20]. A few research groups have elucidated that disulfidptosis plays an important role in various biological processes, such as homeostasis, may be involved in the development of tumors, and may potentially be used for the treatment of tumors [12, 36]. Disulfidptosis represents a mode of cystine accumulation in SLC7A11^high^ cells subjected to glucose starvation and is distinct from other known types of PCDs. The anomalous disulfide bonds between actin cytoskeleton proteins contribute to the collapse of the actin network, which results in alterations in the localization and organization of glycolytic pathway members [12]. Owing to the unique metabolic characteristics of disulfidptosis, ccRCC is more closely associated with disulfidptosis compared to any other type of PCD [37]. In light of this, we conducted an integrative bioinformatics analysis to identify the potential prognostic value of disulfidptosis in ccRCC and disulfidptosis-related targets for ccRCC therapy.

In the present study, we observed that most disulfidptosis-related molecules (9/10) were differentially expressed in ccRCC compared with normal tissues, suggesting a strong correlation between disulfidptosis and ccRCC progression. Based on the expression profiles of disulfidptosis-related molecules, the samples from the comprehensive cohort of TCGA–ccRCC, RECA-EU, and E-MTAB-1980 were classified into three clusters via unsupervised clustering. We then evaluated the levels of TIICs using the ssGSEA method and observed significant differences in the relative abundance of immune cells across the three subgroups. In particular, in DScluster B, the majority of the cells exhibited a notable decrease in immune cell infiltration compared with the other two DSclusters. Unexpectedly, however, DScluster B exhibited the highest count of active CD8 + T cells. In a study of melanoma, enhanced CD8 + T cell infiltration in TME exhibited a correlation with shorter OS [38]. In addition, the extent of CD8 + T cell infiltration in tumor tissues is consistent with the mutation and evolution of tumor cells, thereby contributing to tumor immune escape. To a certain extent, this also elucidates the inferior prognosis of patients in DScluster B. To analyze the downstream genes of the disulfidptosis, we conducted a differential analysis for the three DSclusters and identified 7,740 DEGs. WGCNA revealed 267 hub genes in the tan and salmon modules that were highly correlated with KIR. Enrichment analysis showed that hub genes were associated with the microtubule cytoskeleton and various signaling pathways, such as cell cycle, cellular senescence, and the p53 signaling pathway [12]. The close association of cell cycle and cellular senescence with cell death is well established [39, 40]. In ccRCC, the p53 pathway has been found to exhibit recurrent mutations [41]. We then determined two new gene clusters using the 267 hub genes. The results revealed that geneCluster B exhibited the worst prognosis. These findings provided more insights into the potential molecular mechanisms of disulfidptosis that may have therapeutic implications.

To quantify the status of disulfidptosis in individuals, we developed a scoring system, namely the DSscore system, using prognostic genes to define distinct disulfidptosis groups, thereby elucidating a means to predict the prognosis of ccRCC and guide clinical decision-making with increased accuracy. We calculated DSscores by PCA, and based on the optimal DSscore values, we classified patients with ccRCC into two groups, namely the low- and high-DSscore groups. The patients in the high-DSscore group exhibited particularly significant prolonged OS. Moreover, a significantly positive correlation was observed between the DSscores and most immune cells, consistent with the results of previous studies. For example, T cells constitute the predominant immune cell population within the TME, and their activity ratio is more strongly associated with the effective anti-tumor response in ccRCC [42]. Many studies have elucidated that decreased T cells might reflect the increased risk of ccRCC recurrence [43]. Research has elucidated a positive correlation exists between the proportion of NK cells in ccRCC and a favorable prognosis [44]. Moreover, a few patients with metastatic ccRCC have been observed to not respond to systemic cytokine treatment, which may be attributed, to a notable extent, to the lack of NK cell activation [45]. Generally, plasmacytoid dendritic cells are thought to promote cancer progression and tumor-induced tolerance [46]. The differences in outcomes between different DSscore groups are probably due to the distinct patterns of immune cell abundance. Thus, the potential mechanism of disulfidptosis affecting the progression of ccRCC may be associated with the regulation of immune cell infiltration.

Multiple clinical studies of solid tumors have elucidated that TMB levels may be a promising marker of immunotherapy response, with high TMB indicating better clinical response and longer OS [47–49]. For example, a higher level of TMB has been shown to be associated with a higher probability of a positive response to immune checkpoint inhibitors in patients with lung cancer via targeted next-generation sequencing [49]. In this study, patients in the high-DSscore group exhibited increased TMB, which is likely to be associated with longer survival outcomes. To validate our conjecture, we then explore the prognostic value of DSscore in two cohorts that received immune checkpoint therapy (IMvigor210 and GSE78220 cohorts). Following treatment, patients in the high-DSscore group exhibited significantly longer OS than those in the low-DSscore group, revealing that immunotherapy was one of the appropriate and clinically applicable anticancer strategies for patients with a high DSscore.

Drug susceptibility analysis showed that patients in the low-DSscore group were more sensitive to MIRA-1, navitoclax, linsitinib, and PRIMA-1MET compared with those in the high-DSscore group. The dysregulation of the p53 pathway results in the uncontrolled proliferation of tumor cells. Thus, the resumption of p53 functions, especially p53-dependent apoptosis, is a highly promising approach to anticancer treatment [50]. In the past years, several small molecules that target mut-p53-reactivation, including MIRA-1 and PRIMA-1MET, have been developed [51, 52]. Joerger and Fersht elucidated that MIRA-1 induces p53-independent apoptosis in multiple myeloma cells [51]. Recently, PRIMA-1MET was shown to limit the growth of colorectal cancer cells, irrespective of their p53 status [52]. Navitoclax, a member of the B-cell lymphoma 2 (BCL-2) family of protein inhibitors, was shown to induce apoptosis in cancer cells by disrupting the interactions between BCL-2-like proteins and BH3 domains [53]. The efficacy of navitoclax in the treatment of lung cancer and various relapsed or refractory lymphoid tumors has been elucidated by several phase I clinical studies [53, 54]. As an inhibitor of the insulin-like growth factor 1 receptor (IGF-1R), linsitinib has been identified as a satisfactory chemotherapeutic agent for multiple tumors [55]. Excitedly, in a recent study, the inhibition of IGF-1R using linsitinib reversed oncogenic function caused by the loss and/or downregulation of methylthioadenosine phosphorylase in RCC [56]. These drugs exerted an obvious inhibitory effect on tumor growth and are undoubtedly a promising therapeutic option for the patients in the low-DSscore group.

Finally, by performing qRT-PCR, we validated that the expression of GYS1 and SLC7A11 were upregulated, whereas those of NCKAP1, NDUFS1, NUBPL, OXSM, RPN1, and SLC3A2 were downregulated in ccRCC compared with those in the para-carcinoma tissues. Furthermore, we used scRNA-seq data from seven ccRCC samples to analyze cellular heterogeneity. The results also validated the possibility of disulfidptosis-related molecules in discriminating cell subtypes.

This study involved a comprehensive analysis of the association between disulfidptosis and ccRCC. However, several inevitable limitations existed. First, this study was conducted retrospectively using public databases, which may introduce a certain offset in the data. Second, we investigated the relationship between disulfidptosis and TME in ccRCC; however, we did not elucidate the underlying mechanisms by which disulfidptosis affects immune cells. Third, although qRT-PCR was performed to examine the expression of disulfidptosis-related molecules, additional *in vitro* or *in vivo* experiments are required to elucidate the mechanisms underlying disulfidptosis in ccRCC.

## CONCLUSION

In summary, in this study, we elucidated three distinct disulfidptosis clusters in ccRCC and established a disulfidptosis scoring system for individuals. DSscore grouping has the potential to predict survival outcomes of patients and distinguish immune and clinicopathological characteristics. We also determined the therapeutic liability of DSscore in immunotherapy and chemotherapy; however, further studies are required to validate our findings. Overall, our findings provide a basis and elucidate the potential predictive value of the possible mechanisms underlying the correlation between disulfidptosis and ccRCC. Our findings may contribute to the development of novel clinical interventions.

## Supplementary Materials

Supplementary Figures

Supplementary Table 1

Supplementary Table 2
