# Supplementary material for: Disulfidptosis status influences prognosis and therapeutic response in clear cell renal cell carcinoma
Source: Aging (Albany NY). 2024 Jan 24;16(2):1249–75. doi: 10.18632/aging.205405 (PMC10866437; doi:10.18632/aging.205405)
Supplement: Supplementary Figures [file aging-16-205405-s001.pdf]

SUPPLEMENTARY FIGURES

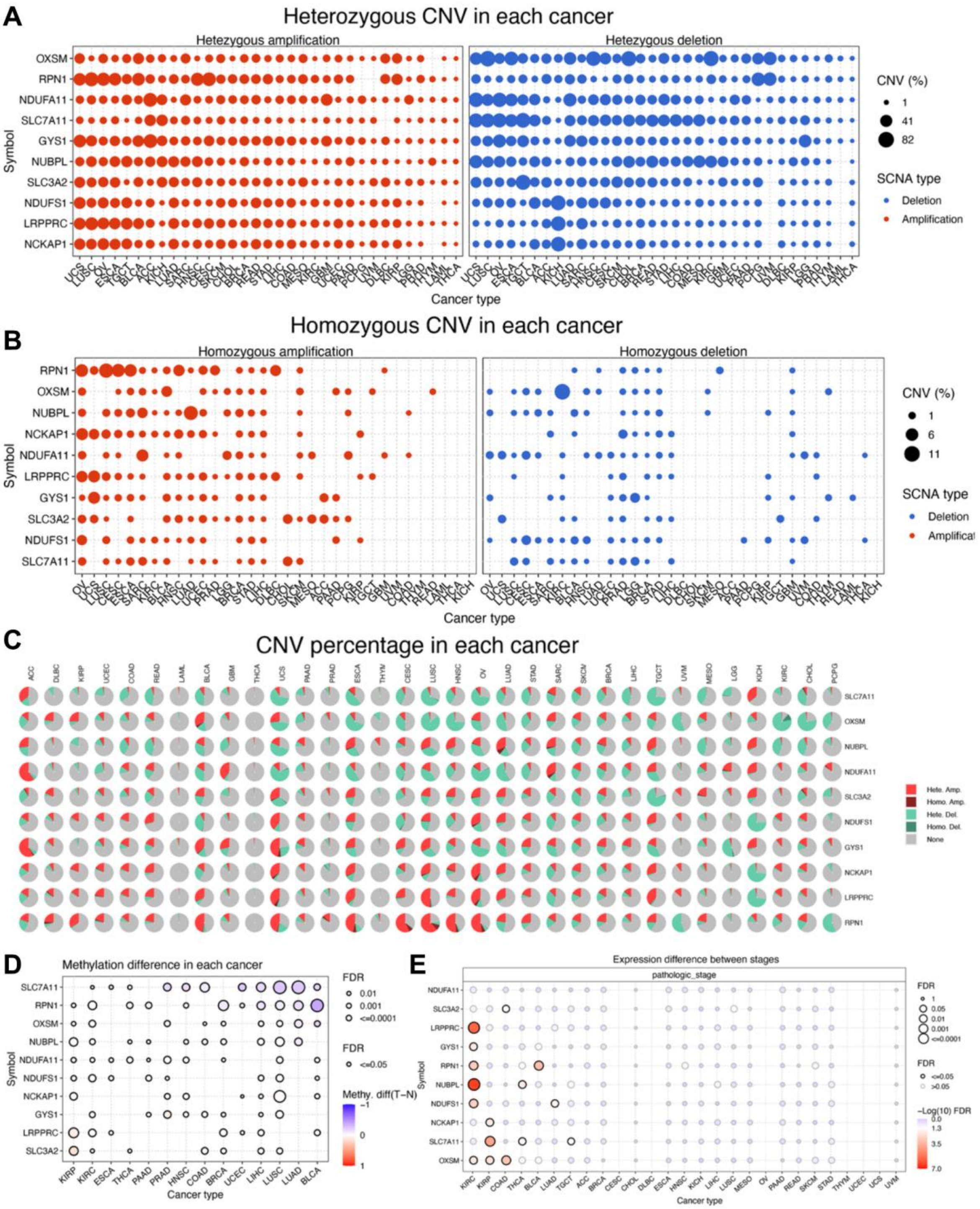

Supplementary Figure 1. Pan-cancer analysis of genetic changes in disulfidptosis-related molecules. (**A**, **B**) A CNV bubble chart showing the distribution of (**A**) heterozygous and (**B**) homozygous CNV of disulfidptosis-related molecules in each type of cancer. (**C**) A CNV pie distribution showing the constitution of heterozygous/homozygous CNV of disulfidptosis-related molecules in each type of cancer. (**D**) A bubble chart showing the differences in methylation of disulfidptosis-relayed molecules between normal and tumor tissues. (**E**) A bubble chart showing the correlation between tumor stage and mRNA expression levels.

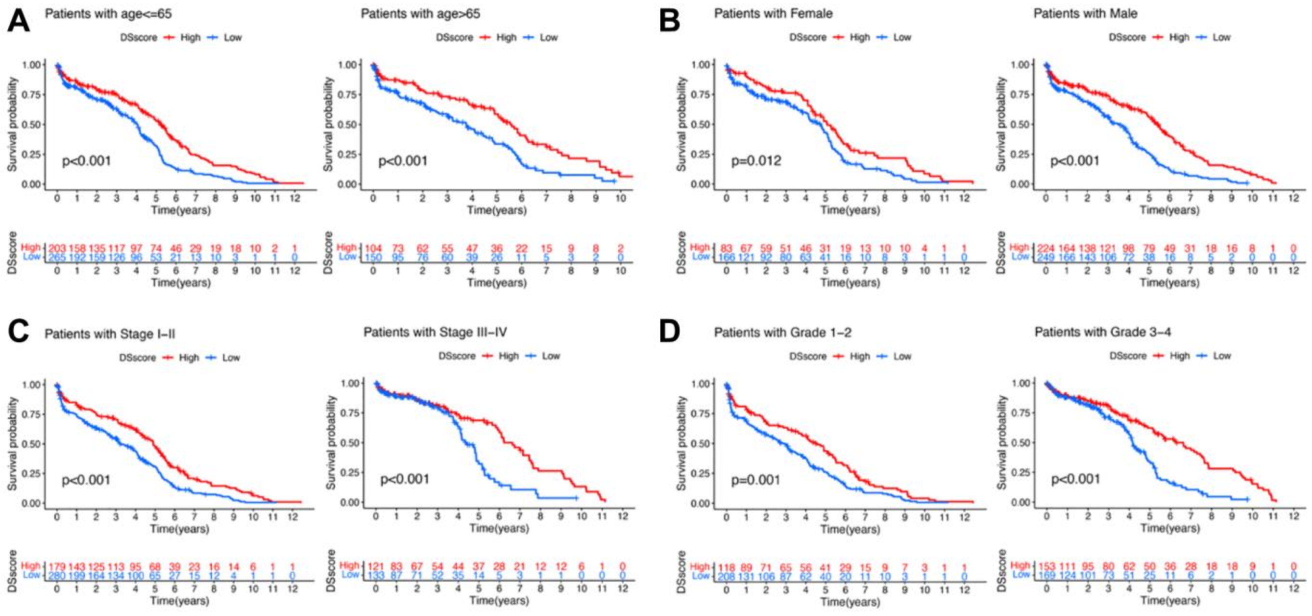

Supplementary Figure 2. Clinical evaluation of the DSscore. Survival analysis of various DSscore groups of patients with KIRC based on age (**A**), sex (**B**), grade (**C**), and (**D**) stage.

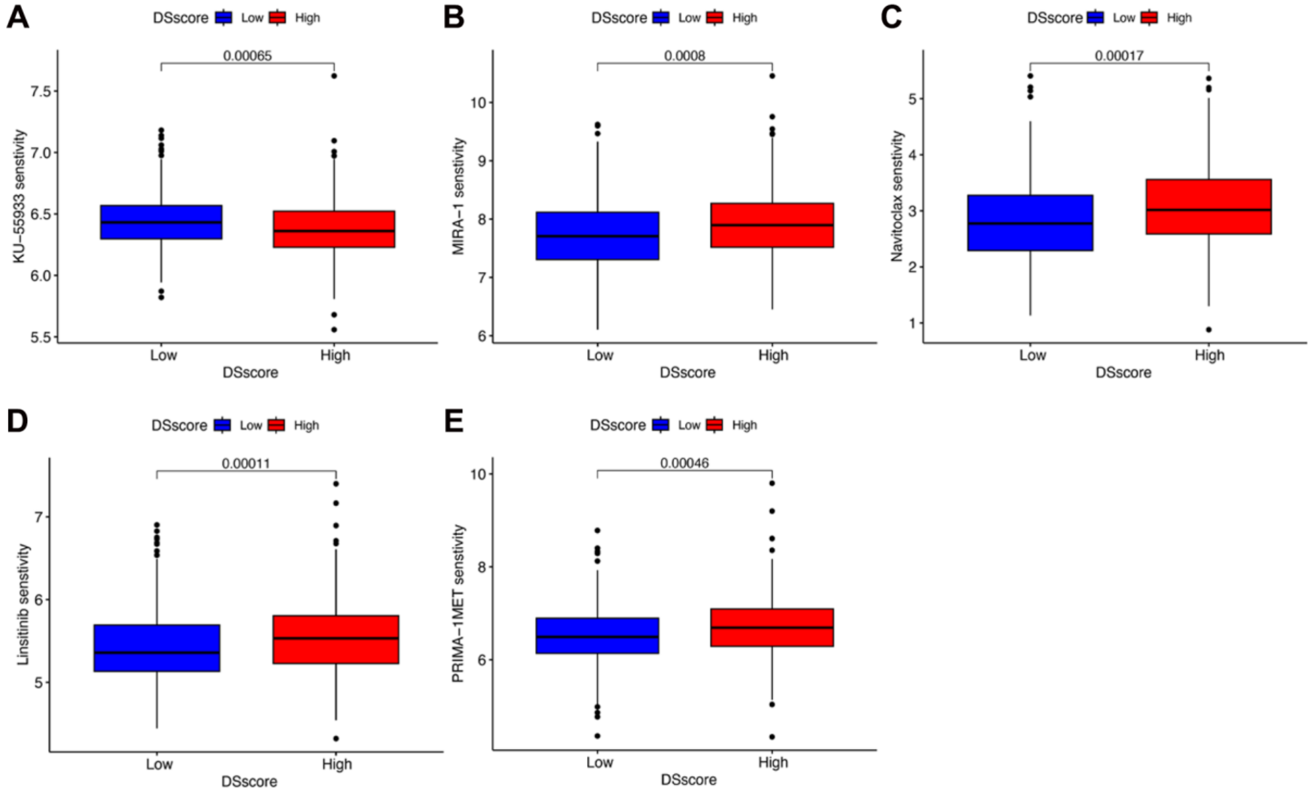

Supplementary Figure 3. Drug sensitivity analysis of the low- and high-DSscore groups. (**A**) KU-55933. (**B**) MIRA-1. (**C**) Navitoclax. (**D**) Linsitinib. (**E**) PRIMA-1MET.

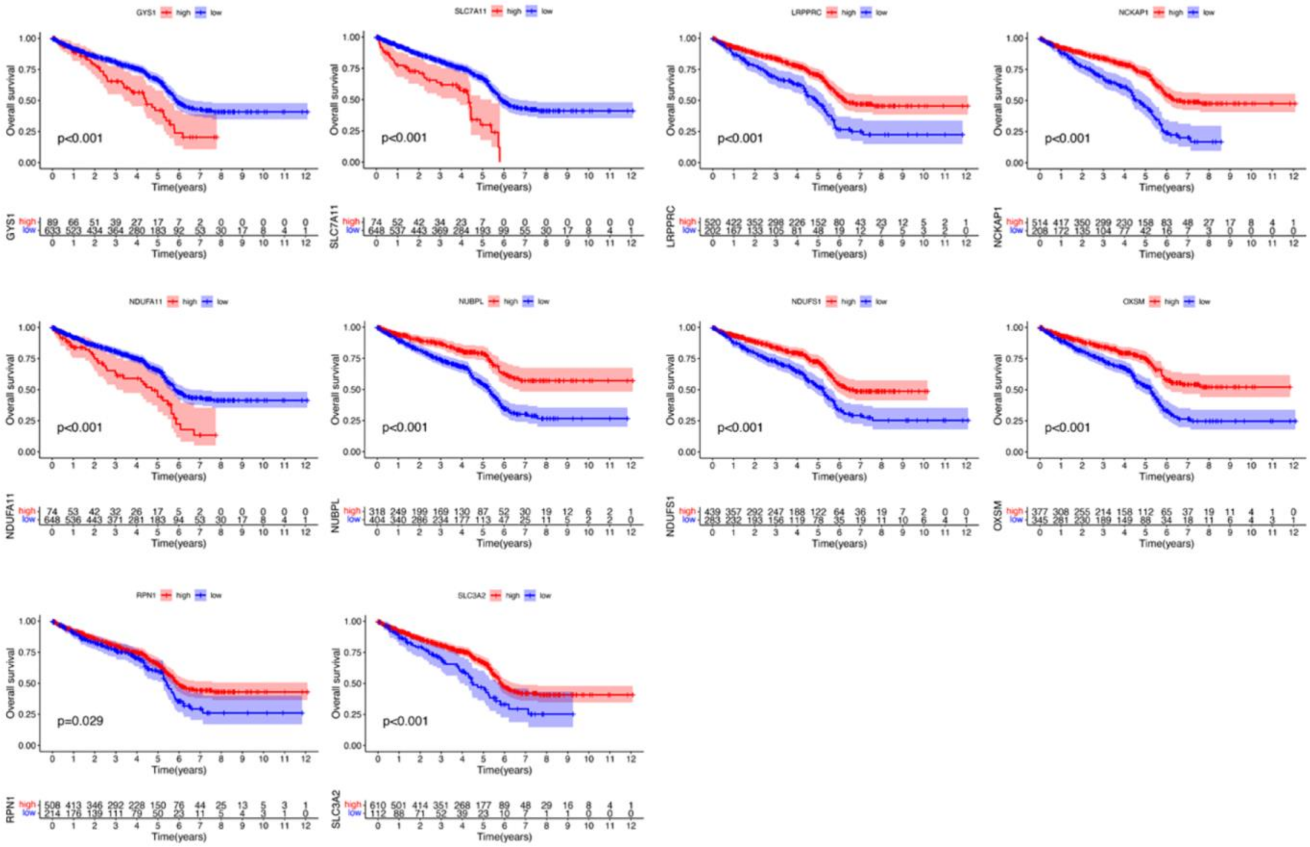

Supplementary Figure 4. Kaplan–Meier survival curves of 10 disulfidptosis-related molecules in the low- and high-DSscore groups.

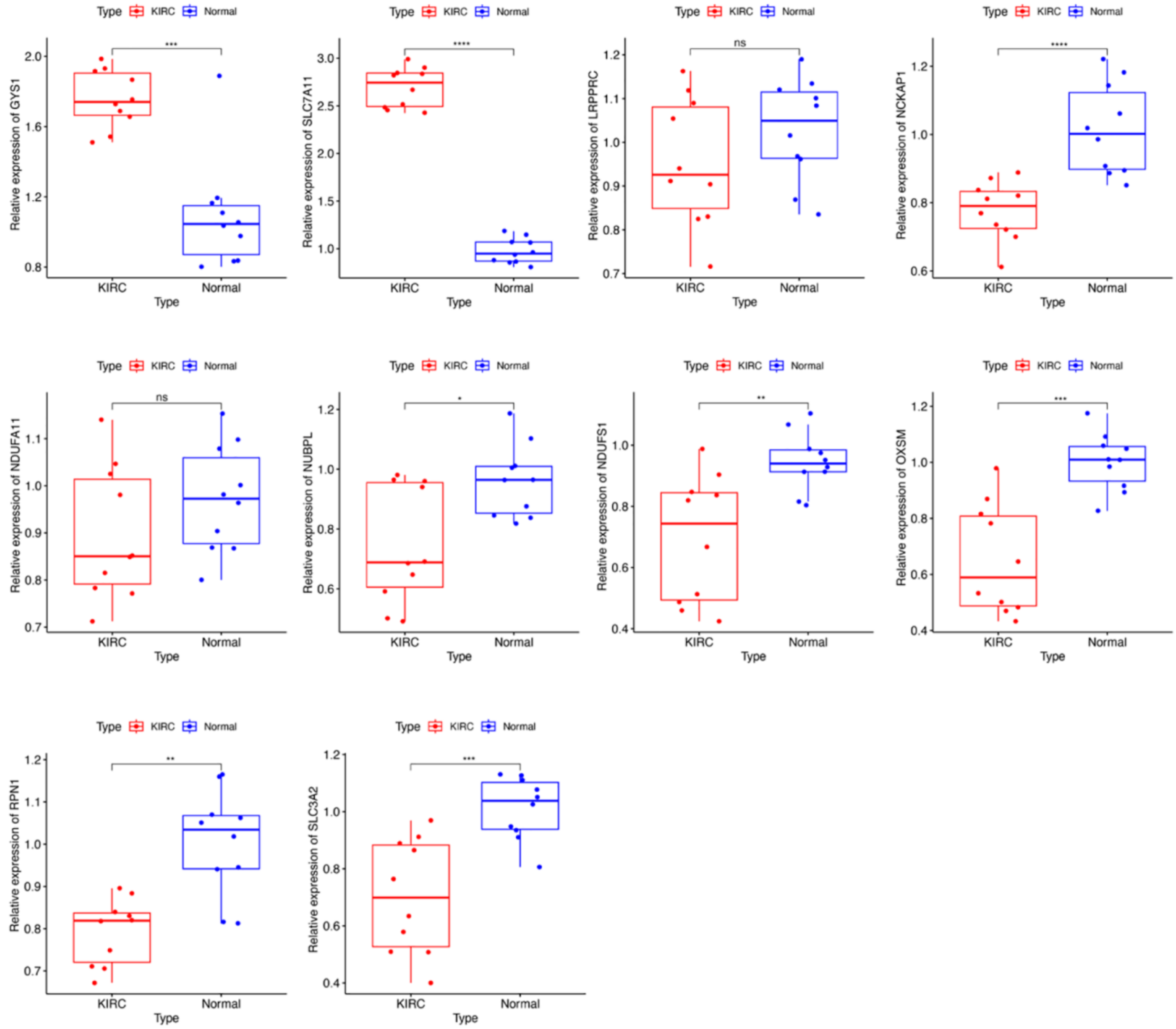

Supplementary Figure 5. Expression levels of 10 disulfidptosis-related molecules in KIRC tissues and corresponding normal tissues as revealed by qRT-PCR.

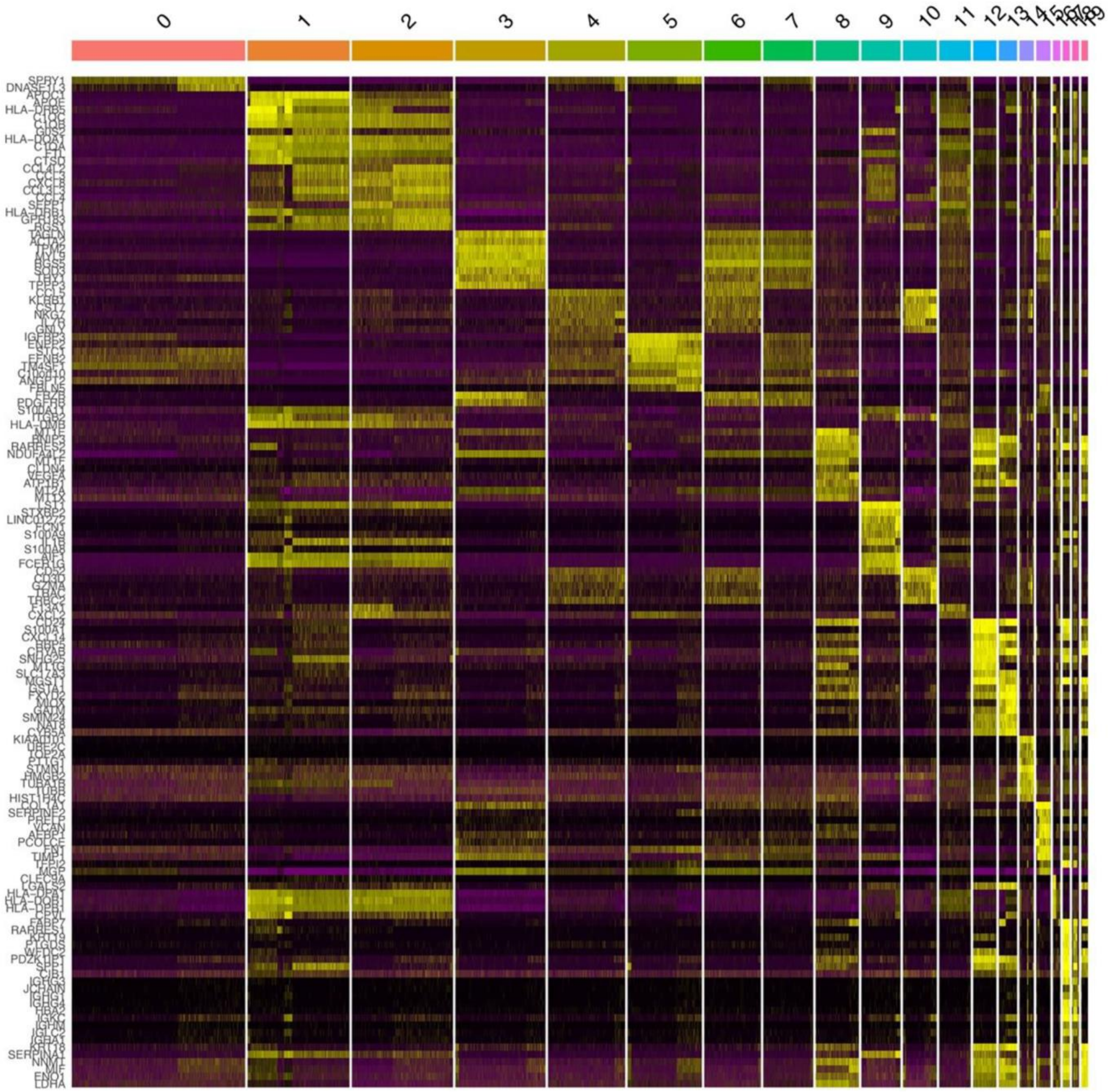

Supplementary Figure 6. Expression analysis of identified marker genes and the top 10% visualized on a heat map.
