# Supplementary material for: Disulfidptosis status influences prognosis and therapeutic response in clear cell renal cell carcinoma
Source: Aging (Albany NY). 2024 Jan 24;16(2):1249–75. doi: 10.18632/aging.205405 (PMC10866437; doi:10.18632/aging.205405)
Supplement: Supplementary Table 1 [file aging-16-205405-s002.pdf]

SUPPLEMENTARY TABLES

Supplementary Table 1. The primers for qRT-PCR.

Genes	Forward primer	Reverse primer
GYS1	5′-TCTCTGCCATGCATGAGTTC-3′	5′-GCCGGCGATAAAGAAATACA-3′
LRPPRC	5′-TTCAGTGCTCTCGTCACAGG-3′	5′-GTCGCGGTCCATGAAGTAAT-3′
NCKAP1	5′-AGATTGATCCTGCATTGGTCGTAGC-3′	5′-ACTGCCACAAACACCATGAGAAGG-3′
NDUFA11	5′- GCCGAAGGTTTTTCGTCAGTA-3′	5′-GGAGGATTGAGTGTGACTCTGT-3′
NDUFS1	5′-TTTCTAGAGGGGAAGCGTGC-3′	5′-TGCAGCGGGTACACTGTATG-3′
NUBPL	5′-CTGAGATGTTTCGCAGAGTCC-3′	5′-CAAGGGTCTGTGCTAGTTTCC-3′
OXSM	5′-CAATATCCAGATTGCATAGGCGA-3′	5′-CGATCCCAAACCAGGTGAGTT-3′
RPN1	5′-TGGTTAAGGTTGGATAAAAGGTATT-3′	5′-ATTTCCCCAATAATAAACAAAACCT-3′
SLC3A2	5′-CTGGTGCCGTGGTCATAATC-3′	5′-GCTCAGGTAATCGAGACGCC-3′
SLC7A11	5′-TGCTGGGCTGATTTTATCTTCG-3′	5′-GAAAGGGCAACCATGAAGAGG-3′
GAPDH	5′-ATCATCCCTGCATCCACT-3′	5′-ATCCACGACGGACACATT-3′
