# Supplementary material for: Disulfidptosis status influences prognosis and therapeutic response in clear cell renal cell carcinoma
Source: Aging (Albany NY). 2024 Jan 24;16(2):1249–75. doi: 10.18632/aging.205405 (PMC10866437; doi:10.18632/aging.205405)

**Supplementary Table 2. The survival data of ccRCC patients included in this study and the distribution of DScluster, gencluster, and DSscore.**


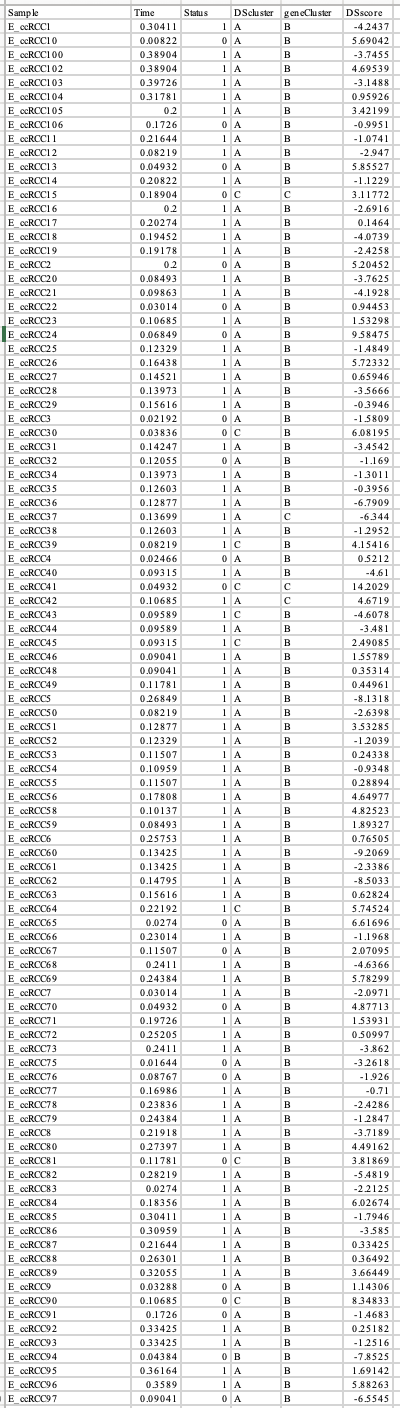

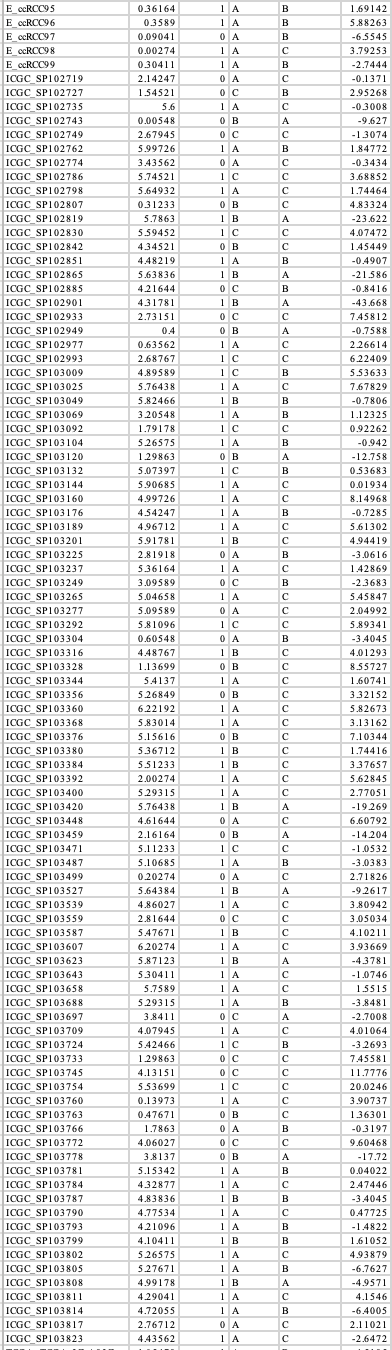


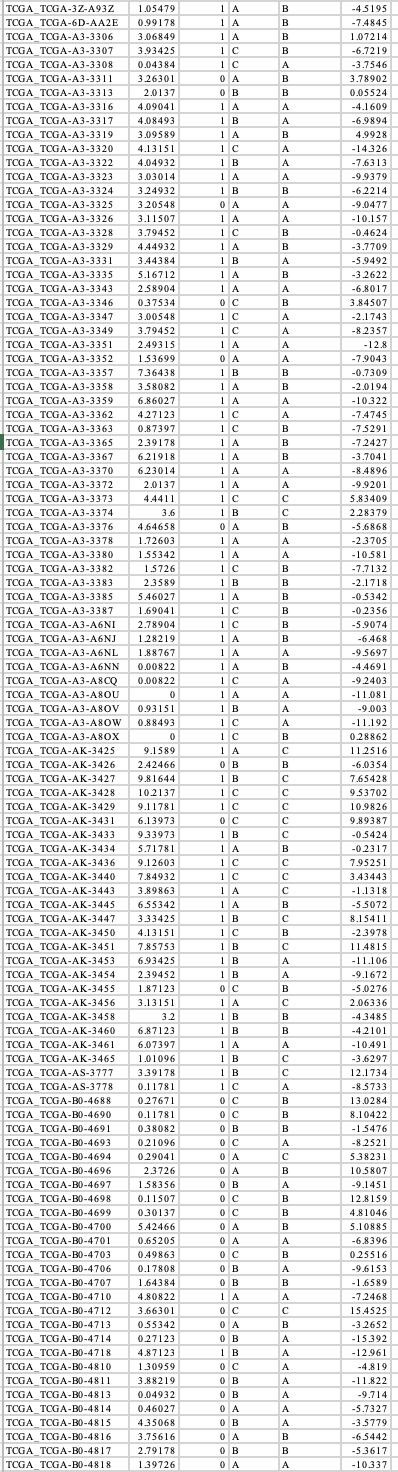

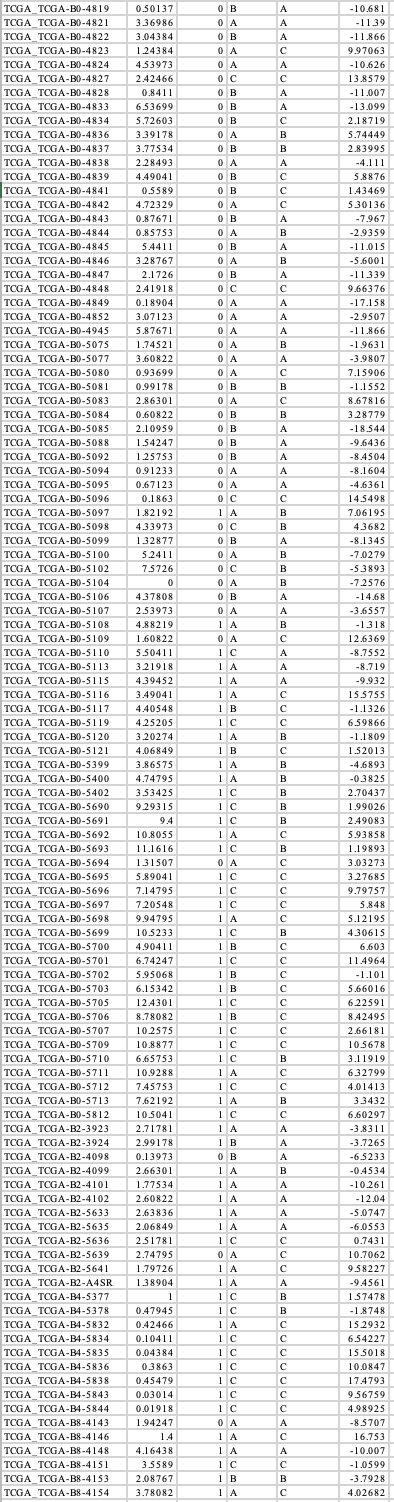


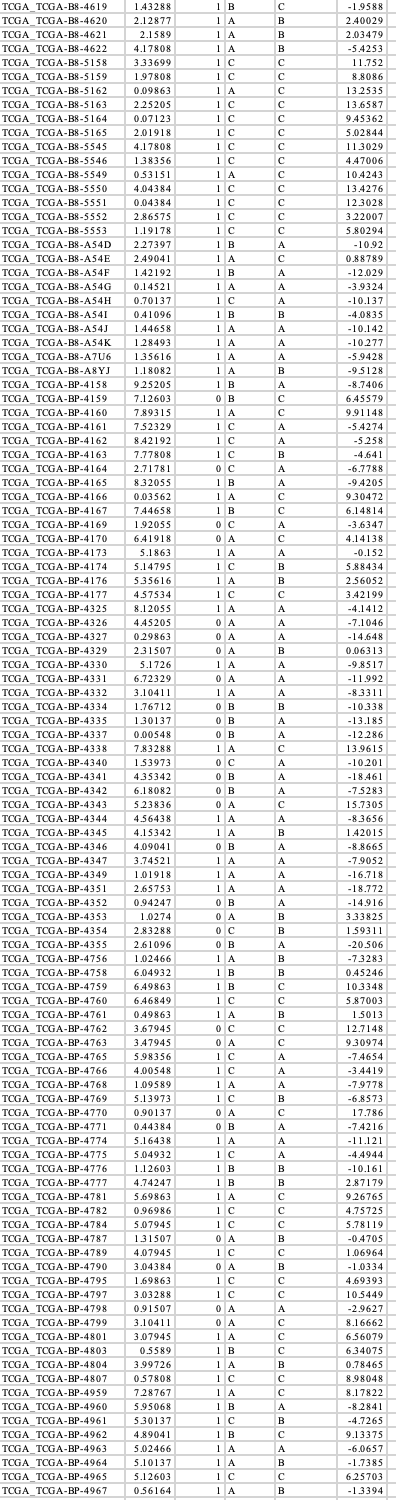

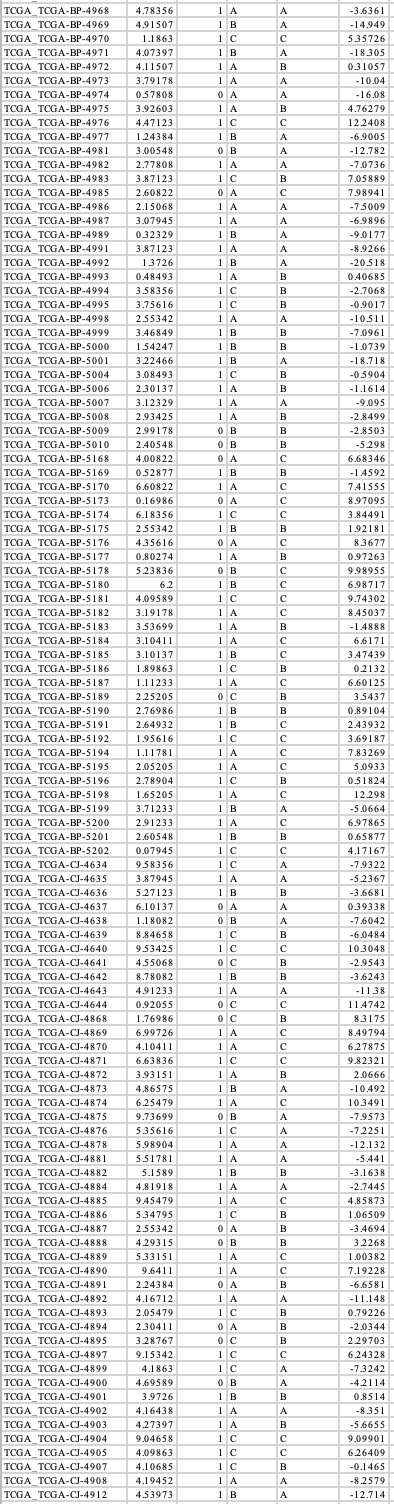


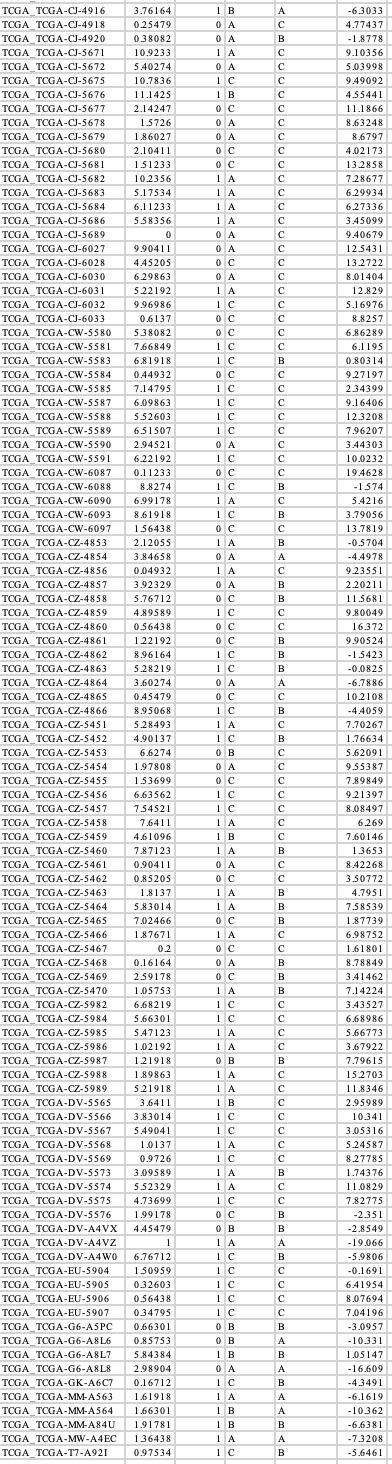

Supplement: Supplementary Table 2 [file aging-16-205405-s003.docx]
